# Pocket-Surface Discrete Differential Geometry as a Leakage-Robust Feature Class for Protein–Ligand Binding Affinity Prediction

**DOI:** 10.3390/molecules31111899

**Published:** 2026-06-01

**Authors:** Mehmet Ali Balcı, Erbil Çetin, Gizem Calibasi-Kocal, Ömer Akgüller

**Affiliations:** 1 Department of Mathematics, Faculty of Science, Mugla Sitki Kocman University, 48000 Mugla, Turkey; mehmetalibalci@mu.edu.tr; 2 Department of Mathematics, Faculty of Science, Ege University, 35100 Izmir, Turkey; erbil.cetin@ege.edu.tr; 3 Translational Oncology Department, Oncology Institute, Dokuz Eylul University, 35340 Izmir, Turkey; gizem.calibasi@deu.edu.tr; 4 Oncology Department, Institute of Health Sciences, Dokuz Eylul University, 35340 Izmir, Turkey

**Keywords:** protein–ligand binding affinity, structure-based drug discovery, discrete differential geometry, Laplace–Beltrami operator, heat-kernel signature, molecular surface, leakage-protected splits

## Abstract

Protein–ligand binding affinity prediction underpins structure-based drug discovery, yet random partitions of public benchmarks overestimate generalisation due to protein-family and ligand leakage, and the marginal value of explicit pocket-geometry descriptors over atom-level graph neural networks remains unclear. We computed a 59-dimensional discrete differential geometry descriptor on the ligand-aware solvent-excluded surface of 3285 PDBBind v2020 complexes, combining curvature distributions, the leading sixteen Laplace–Beltrami eigenvalues and a ten-point heat-kernel signature, and evaluated it in gradient-boosted tree pipelines across progressively stricter split regimes and two leak-proof external benchmarks, together with four mechanistically distinct injection strategies in a SchNet-style graph neural network. The descriptor lifted Pearson correlations by 0.111 on cluster-disjoint testing, 0.258 on LP-PDBBind DataSAIL S2 and 0.365 on CASF-2016, while in isolation reaching 0.456 to 0.594 on external benchmarks, on a par with X-Score and AutoDock Vina (version 1.2). TreeSHAP attribution localised the dominant signal to the heat-kernel signature. The four graph neural network injection strategies produced no statistically significant lift, indicating that distance-based message passing on atomic coordinates already captures much of the geometric content. Pocket-surface discrete differential geometry, therefore, offers an interpretable, leakage-robust and lightweight feature class for early-stage virtual screening, and motivates hybrid mesh-to-atom architectures.

## 1. Introduction

The accurate ranking of small-molecule binding affinities to protein targets sits at the centre of structure-based drug discovery [1,2,3]. Across virtual libraries that now routinely exceed billions of compounds, the difference between a tractable lead-optimisation campaign and a wasted experimental cycle often reduces to the question of whether a scoring function can correctly order candidate molecules by their experimental dissociation or inhibition constants. The classical empirical scoring functions that dominated the field for two decades, exemplified by AutoDock Vina [4] and X-Score [5], combine weighted physicochemical terms in an additive functional form and plateau at Pearson correlations of about 0.60 on the CASF-2016 scoring power benchmark of Su et al. [6]. Their accuracy is bounded by the expressiveness of the analytic form rather than by the data, and the ceiling has resisted incremental refinement. A complementary and more physically grounded family of methods estimates binding free energies from molecular dynamics endpoints rather than from a single docked pose, most prominently the molecular mechanics Poisson–Boltzmann and generalised Born surface area approaches, which provide an explicit account of solvation and configurational averaging at a markedly higher computational cost [7].

Over the past five years, atom-level graph neural networks have displaced these classical scoring functions as the de facto state of the art. By representing the protein–ligand complex as a graph whose nodes are heavy atoms and whose edges are distance-based or contact-based, models such as InteractionGraphNet [8], the geometric interaction network GIGN [9], the interaction-based inductive bias framework EHIGN [10], the multi-objective network PLANET [11], and the physics-informed PIGNet2 [12] have advanced the state of the art to Pearson correlations of approximately 0.84 on CASF-2016 and to roughly 0.50 on the leakage-protected LP-PDBBind partitions reported by Li et al. [13]. The field is consolidating around the position that geometric inductive biases, when supplied through three-dimensional message passing, deliver substantial gains over sequence and ligand-only representations [14,15,16,17].

Despite this consolidation, two tensions continue to shape methodological work in protein–ligand affinity scoring. The first is the problem of dataset leakage, which the field has only recently learned to take seriously. The audit by Volkov et al. [18] showed that random partitions of PDBBind overestimate held-out performance by approximately 0.20 in Pearson correlation relative to target-disjoint splits, an inflation attributable to ligand similarity and protein-family memorisation rather than to learned chemistry. Bernett et al. [19] later formalised this into a set of guiding questions that any biological machine learning benchmark must answer before its reported performance can be trusted. In response the community has converged on progressively stricter evaluation protocols, including target clustering at 30% sequence identity, ligand-similarity cutoffs, the LP-PDBBind splits of Li and colleagues, and most recently the optimisation-based DataSAIL splits of Joeres et al. [20], in which a binary linear program directly minimises the residual information leakage between training and test partitions. The second tension concerns the chemical interpretability of the features that drive prediction. Distance-based message passing on atomic coordinates is sufficiently expressive that it is often unclear whether additional, hand-crafted geometric descriptors of the binding pocket carry information that is genuinely complementary to what three-dimensional atomic graphs already encode, and unclear where in the pipeline such descriptors contribute most.

Discrete differential geometry, as developed in the applied mesh processing literature [21,22], provides a principled vocabulary for describing the binding pocket as a triangulated two-manifold and extracting rotation-invariant, translation-invariant and approximately isometry-invariant descriptors of its shape. Mean and Gaussian curvatures characterise local convexity and topological deviation, the principal curvatures and the derived shape index resolve saddle, ridge and bowl geometries, the leading eigenvalues of the discrete Laplace–Beltrami operator constitute the so-called shape DNA of the surface and encode its global spectrum in the sense of Reuter et al. [23], and the heat-kernel signature of Sun et al. [24] provides a multi-scale descriptor by sampling the diagonal of the heat kernel at several diffusion times. Short diffusion times reveal local curvature, while longer times report on cavity dimension and global enclosure. These descriptors have been profitably used in protein-protein interaction studies through MaSIF and its differentiable extension dMaSIF [25,26], and surface representations have very recently been combined with multimodal encoders to predict binding affinities [27]. Curvature-aware graph neural networks such as CurvAGN [28] have shown that pocket geometry can be exploited inside three-dimensional message-passing architectures, and the broader programme of topological data analysis has produced a family of persistent homology, persistent Laplacian and Mayer homology descriptors for binding affinity that reach state-of-the-art accuracy on standard benchmarks [29,30]. What has not been quantified, to our knowledge, is the direct utility of a fixed-size, interpretable discrete differential geometry descriptor of the ligand-aware pocket surface for protein–ligand affinity scoring under the strictest leakage-protected protocols that the community now demands.

The present study addresses this gap. Our first concern is to establish whether a compact discrete differential geometry descriptor of the ligand-aware solvent-excluded pocket surface carries a leakage-robust affinity signal that survives increasingly strict evaluation regimes, up to and including the strictest leak-proof partitions currently available. Our second concern is to localise, through ablation and TreeSHAP attribution, the components of the descriptor that drive that signal, so that the matter-of-fact statement that pocket geometry helps can be refined into a chemically interpretable claim about which geometric modes carry information. Our third concern is to delineate the operational scope of the descriptor by comparing it to representative atom-level graph neural network treatments of the same geometric content. To this end we compute a 59-dimensional descriptor on every PDBBind v2020 refined-set complex with publicly retrievable structures and a parseable mesh (n=3285), benchmark it across four internal split regimes of increasing stringency, including a *k*-mer Jaccard cluster-disjoint protocol at three thresholds, and evaluate it against two external held-out benchmarks, namely the CASF-2016 core set and the leak-proof LP-PDBBind DataSAIL S2 partition. We report bootstrap confidence intervals over 500 resamples, a 5000-iteration paired permutation test, and a five-seed cluster-split ensemble. As a complementary architectural analysis, we further inject the same descriptor into a SchNet-style distance-based graph neural network [31] through four mechanistically distinct strategies, namely global concatenation at the readout stage, per-atom projection at the embedding stage, geometry-conditioned cross-attention, and curvature and heat-kernel-conditioned edge features in the spirit of CurvAGN.

Our principal finding is that pocket-surface discrete differential geometry behaves as a leakage-robust feature class. In gradient-boosted tree pipelines without direct access to atomic coordinates, the descriptor produces consistent and statistically significant gains that grow with split stringency, reaching 0.111 in Pearson correlation on cluster-disjoint testing, 0.258 on the leak-proof LP-PDBBind DataSAIL S2 partition and 0.365 on CASF-2016, while the descriptor used in isolation matches classical scoring-function performance on both external benchmarks. Component ablation and SHAP attribution identify the heat-kernel signature, particularly at short and mid-range diffusion times, as the dominant carrier of the signal, consistent with the physical interpretation of those time scales as probes of local curvature, cavity dimension and the rigidity modes that govern desolvation and binding entropy. The complementary architectural analysis demonstrates that, within a small SchNet-style distance-based backbone, none of the four injection strategies exceeds a Pearson lift of 0.02 on cluster-disjoint testing, with a mean lift of approximately 0.004. Read together with the strong feature-based effect, this absence of lift is best understood not as evidence that the geometric content is uninformative but as evidence that distance-based message passing on atomic coordinates already captures much of it, leaving an architectural frontier for networks that treat the pocket surface as a first-class object rather than as a collection of atom-anchored projections.

The practical implication is that pocket-surface discrete differential geometry is well suited to the lightweight, interpretable and computationally efficient pipelines used in early-stage virtual screening and in resource-limited settings, where it can recover classical scoring-function performance from a 59-dimensional descriptor while remaining inspectable by a medicinal chemist. At the same time, our results motivate a research direction in which ligand atoms attend directly to the spectral and curvature information of the pocket surface through hybrid mesh-to-atom architectures, extending the surface-based geometric deep learning programme that has so far been most successful in protein-protein interaction prediction to the protein–ligand affinity setting.

## 2. Results

**Figure 1:**
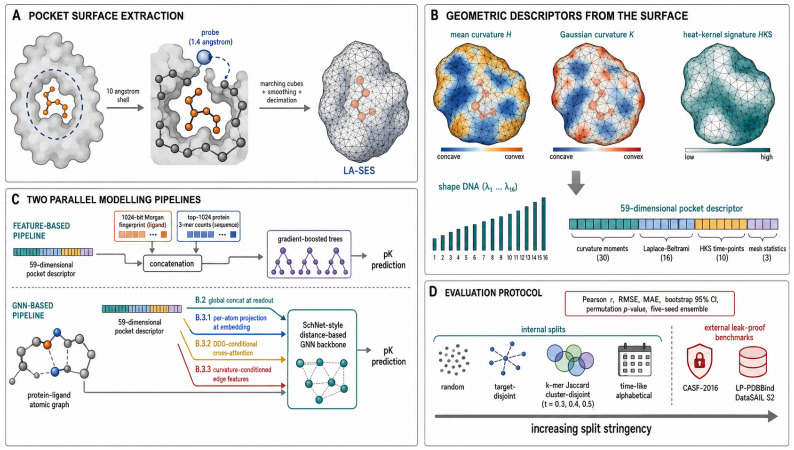
Overview of the analysis workflow. (**A**) Construction of the ligand-aware solvent-excluded surface (LA-SES) from the protein–ligand complex. The dashed blue circle marks the 10 Å shell around the ligand within which protein heavy atoms are retained, and the small blue sphere denotes the 1.4 Å solvent probe rolled over those atoms; black arrows denote the sequential processing steps from pocket extraction through marching cubes, smoothing and decimation to the final triangulated LA-SES. (**B**) Extraction of per-vertex geometric quantities and spectral features that compose the 59-dimensional pocket descriptor. The blue-to-orange and blue-to-red bipolar colour scales encode concave-to-convex variation of the mean and Gaussian curvatures respectively, the teal scale encodes low-to-high heat-kernel signature values, and the four colours of the descriptor bar correspond to the curvature moments (30 dimensions), Laplace–Beltrami eigenvalues (16), heat-kernel signature time-points (10) and mesh statistics (3) groups. (**C**) Two parallel modelling pipelines, namely a feature-based gradient-boosted tree regressor that concatenates the descriptor with ligand fingerprint and protein 3-mer features, and a SchNet-style graph neural network into which the descriptor is injected through four mechanistically distinct strategies; the four coloured arrows labelled B.2, B.3.1, B.3.2 and B.3.3 distinguish these injection points. The ellipses inside the fingerprint and 3-mer count blocks indicate unshown intermediate dimensions of these high-dimensional vectors and do not affect interpretation. (**D**) Evaluation protocol across four internal splits of progressively increasing stringency and two external leak-proof benchmarks.

Figure 1 summarises the four-stage analysis workflow developed in this study, from the construction of the ligand-aware pocket surface and the extraction of its geometric descriptors, through the two parallel modelling pipelines we evaluated, to the leakage-protected splits and external benchmarks used for assessment. The full methodological detail of each stage is given in Section 4.

### 2.1. Dataset Characterisation and a Size-Bias Performance Floor

**Figure 2:**
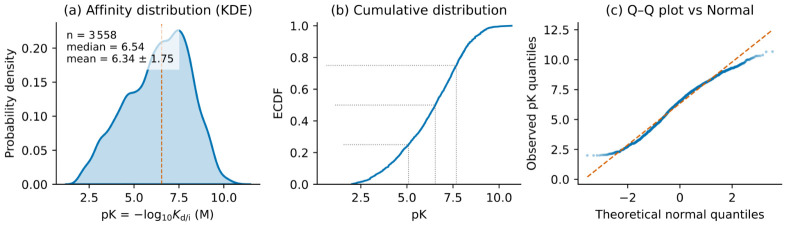
Affinity (pK) distribution of the labelled PDBBind v2020 refined subset (n=3558 complexes after sequence-length, pK-range and uniqueness filtering). The discrete differential geometry analyses use the n=3285 subset for which structures were retrievable and a parseable mesh could be computed. Panel (**a**) shows the kernel density estimate with the median (orange dashed) at pK=6.5; panel (**b**) shows the empirical cumulative distribution function with quartile guidelines; panel (**c**) shows the quantile–quantile plot against the standard normal, indicating a slight negative skew.

**Figure 3:**
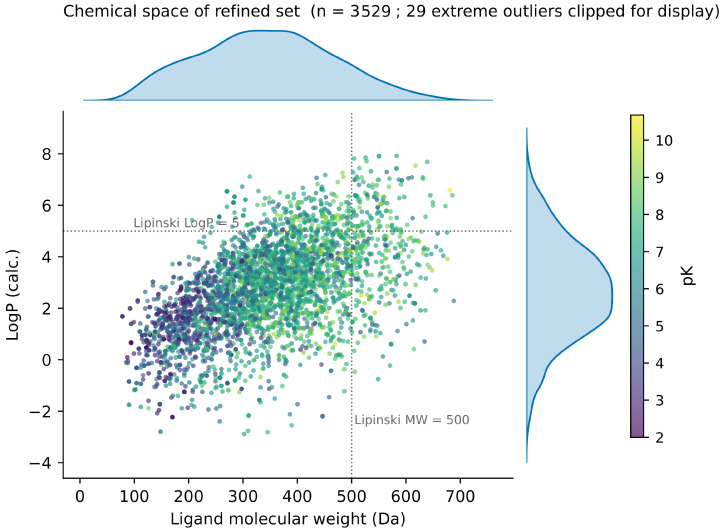
Chemical space coverage of the refined set, plotting calculated LogP against molecular weight, coloured by experimental pK, with marginal kernel density estimates. Lipinski reference lines at MW=500Da and LogP=5 are shown in grey. Of the 3558 ligands, 29 with extreme molecular weight or LogP values were clipped from the display for legibility.

**Figure A1:**
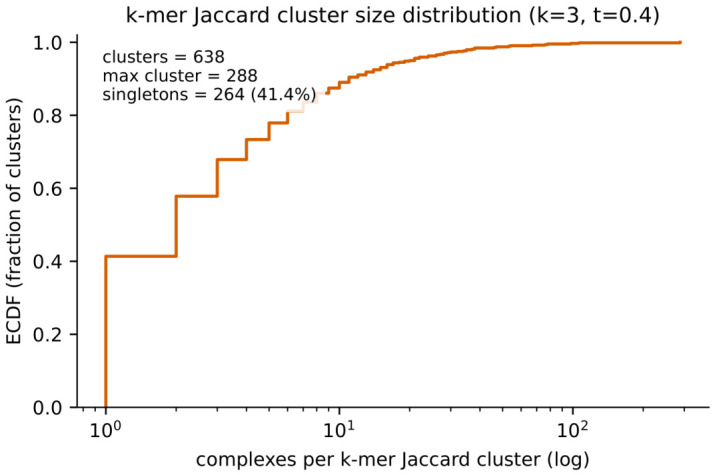
Empirical cumulative distribution of cluster size after *k*-mer Jaccard greedy single-linkage clustering at threshold t=0.4. Of 638 clusters, 264 (41.4%) are singletons and the largest cluster contains 288 complexes corresponding to a kinase-like family.

**Figure A2:**
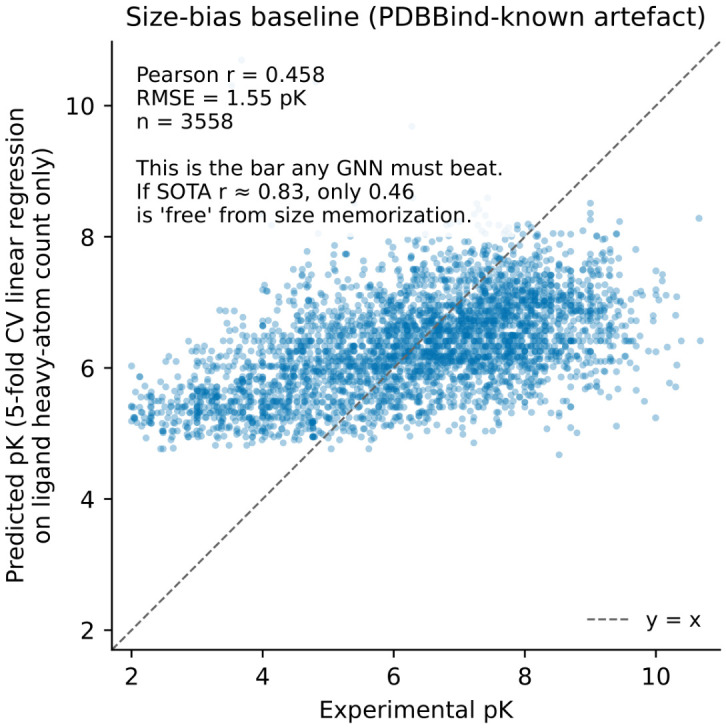
Size-bias baseline. A five-fold cross-validated linear regressor on ligand heavy-atom count alone reaches a Pearson correlation of 0.458 on the full 3558-complex set. Each blue dot represents one protein–ligand complex, with predicted pK (cross-validated linear regression on ligand heavy-atom count) on the vertical axis and the experimental pK on the horizontal axis; the grey dashed line is the identity y=x. This is the free performance available without any chemical or structural information; it is also a known artefact of the PDBBind-Refined collection, and any contribution from the discrete differential geometry block must clearly exceed it under leakage-protected splits.

**Figure A3:**
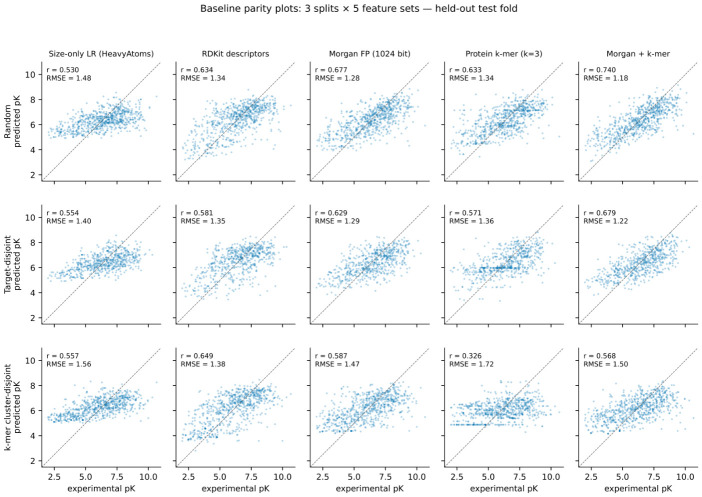
Parity grid for all five baseline feature sets across the three internal split regimes (random, target-disjoint, *k*-mer cluster). Each cell reports the Pearson correlation and the root-mean-square error on the corresponding held-out test fold. The dramatic collapse of the protein 3-mer column from random to cluster split (**top** to **bottom**) is the practical motivation for the analyses reported in the main text.

The 3558 labelled refined-set complexes span an 8.7-pK dynamic range with a median of 6.5 and a slight negative skew (Figure 2). Among the 2240 unique sequences, 81.6% are singletons, while a long tail extends to 120 complexes per target; the empirical cluster size distribution after *k*-mer Jaccard greedy single-linkage clustering at threshold t=0.4 is shown in Figure A1 of Appendix A. To establish a non-cheatable performance floor that any model claiming geometric or chemical insight must clearly exceed, we trained a five-fold cross-validated linear regressor on ligand heavy-atom count alone. This baseline reached a Pearson correlation of 0.458 with a root-mean-square error of 1.55pK on the full 3558-complex set (Figure A2 of Appendix A). The exercise quantifies the free performance available without any chemical or structural information and provides a critical reference point for the leakage-protected analyses that follow. Parity plots for all five baseline feature sets across the three internal split regimes, including the sharp collapse of the protein 3-mer column on the cluster-disjoint split, are provided in Figure A3 of the same appendix. The chemical space coverage of the set is summarised in Figure 3.

### 2.2. Cluster-Disjoint Testing Quantifies Baseline Leakage

**Figure 4:**
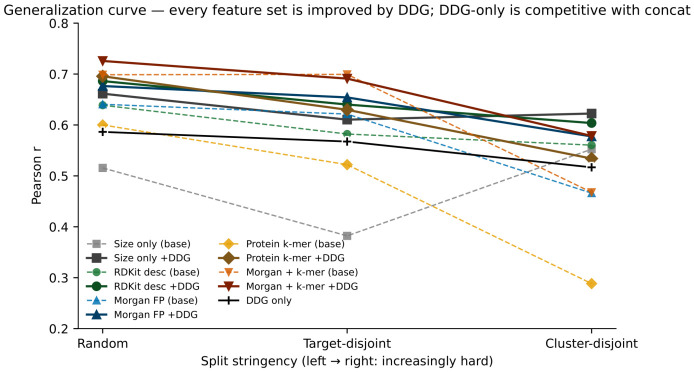
Each line traces the held-out Pearson correlation of one feature set across three split regimes of increasing stringency. Dashed lines correspond to the feature set alone, solid lines to the same feature set augmented with the 59-dimensional discrete differential geometry block; the DDG-only configuration is shown as a solid black line with cross markers. The protein *k*-mer block (yellow dashed) collapses from 0.63 on the random split to 0.29 on the cluster split, but recovers to 0.53 once the geometric block is added (dark olive solid). The DDG-only line remains competitive across all stringency levels.

When the training and test partitions are constrained so that no *k*-mer-similar sequence is shared, with Jaccard similarity at or above 0.4, the concat baseline drops from a random-split Pearson correlation of 0.699 to 0.468. The decrement of 0.23 quantifies the fraction of random-split performance attributable to target memorisation. The protein 3-mer block alone collapses from 0.633 on the random split to 0.288 on the cluster-disjoint split, which confirms that this block functions almost entirely as a memorisation channel rather than as an indicator of binding determinants (Figure 4, the *k*-mer line). The collapse is the practical motivation for our subsequent analysis. A sequence-anchored channel that loses two-thirds of its predictive weight under cluster-disjoint testing cannot be the basis for a deployable scoring system, and any genuinely structural feature class should at a minimum partially fill the resulting gap.

### 2.3. Discrete Differential Geometry Adds a Leakage-Robust Lift in Feature-Based Learners

**Figure 5:**
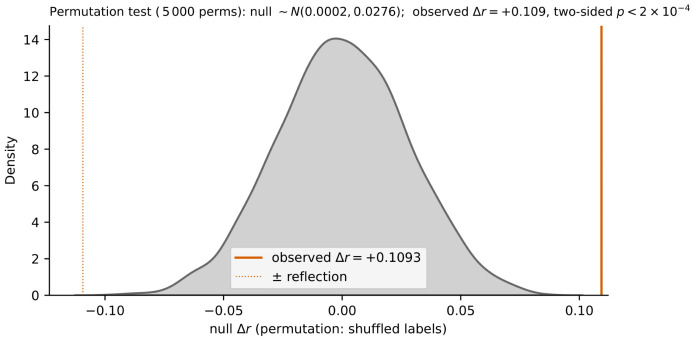
Permutation test for the lift induced by the discrete differential geometry block. The grey distribution is the 5000-iteration null obtained by random swaps of the base and +DDG assignments for each test sample. The orange solid line marks the observed Δr=+0.109 on the cluster-disjoint test fold. The observed value lies outside the support of the null distribution, with two-sided $p<2\times 10^{-4}$ as a conservative upper bound.

**Figure 6:**
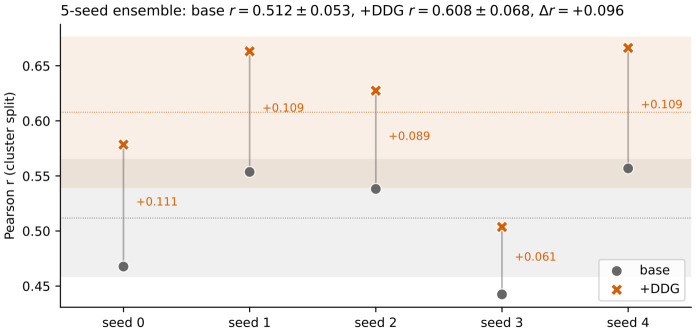
Five-seed ensemble on the cluster-disjoint test fold (t=0.4). Each abscissa position corresponds to one seed configuration (cluster-split seed plus 44, gradient-booster seed between 0 and 4). Grey dots correspond to the concat baseline and orange crosses to the same baseline augmented with the DDG block; vertical grey segments connect the paired predictions per seed and the orange labels give the per-seed Pearson lift. The horizontal grey dashed line marks the seed-mean Pearson correlation of the baseline across the five seeds, and the horizontal orange dashed line marks the seed-mean of the augmented model; the grey and orange shaded bands show ±σ around the corresponding seed-means. The Pearson lift is positive in every seed and clusters around 0.10.

Table 1 reports the cluster-disjoint Pearson correlation for each baseline with and without the discrete differential geometry block. The geometric block produces a positive lift for every baseline. The most informative pairing concerns the protein 3-mer block, where the addition of geometry lifts the Pearson correlation from 0.288 to 0.534, a recovery that almost fully closes the gap opened by cluster-disjoint testing. On the fingerprint-and-3-mer concat baseline, the lift is 0.111, with a permutation test of 5000 iterations yielding $p<2\times 10^{-4}$ (Figure 5). A control in which the 59-dimensional discrete differential geometry block was first residualised against the surface area channel by per-channel linear regression, and only the residuals were concatenated to the baseline, preserves the lift (Figure A8 of Appendix B). The signal is therefore not an artefact of pocket size correlating with ligand size. A five-seed ensemble that varied both the cluster partition assignment and the gradient-booster seed yielded a Pearson lift distribution of mean +0.096 and standard deviation 0.021, with all five seeds returning a positive lift (Figure 6). The 5000-iteration permutation test shown in Figure 5 places the observed Δr=+0.109 entirely outside the support of the null distribution, which is approximately N(0.0002,0.028), with no randomised pairing producing a Δr at least as large as the observed value. A detailed per-baseline view of the marginal contribution of the geometric block across the three internal split regimes is provided in Figure A7 of Appendix B, and parity plots that compare base versus augmented configurations on the cluster-disjoint test fold for all five baseline feature sets are provided in Figure A9 of the same appendix. The full per-configuration metric table for the cluster-disjoint test fold, including 95% bootstrap confidence intervals, root-mean-square errors and mean absolute errors, is reported in Table A2 of Appendix C.

### 2.4. Component Ablation Localises the Signal to the Heat-Kernel Signature

Table 2 summarises an ablation in which each sub-block of the 59-dimensional descriptor is added in isolation to the concat baseline. The dominant contribution comes from the heat-kernel signature, which on its own at ten dimensions yields a Pearson lift of +0.117, exceeding the lift of the full 59-dimensional block at +0.110. Curvature moments at 30 dimensions yield a lift of +0.095, the leading sixteen Laplace–Beltrami eigenvalues yield +0.093 and the three mesh statistics yield +0.094. The sub-additivity of the combined block reflects the mutual redundancy between channels that probe overlapping geometric content.

The ascendancy of the heat-kernel signature admits a physical interpretation. The expansion in Equation (3) weights the contribution of each Laplace–Beltrami mode by an exponential time decay. Short diffusion times probe local curvature in the Gaussian and mean sense and therefore recapitulate information that the curvature features already provide; this is consistent with the ablation result that curvature alone delivers a comparable but smaller lift of +0.095. Mid-range diffusion times correspond to diffusion lengths of approximately one to three Angstroms on the LA-SES mesh, the scale at which ligand atoms encounter the wall of the binding cleft and at which desolvation operates. Long-range times probe the global pocket spectrum, the same shape signature that distinguishes open shallow pockets from deep enclosed ones and that has been shown to correlate with conformational rigidity and hence with the entropic component of binding. The empirical observation that the ten-dimensional heat-kernel signature alone matches or exceeds the lift of the full 59-dimensional descriptor suggests that mid-to-long pocket shape modes carry binding-relevant information that point-wise curvature alone cannot summarise. This finding aligns with the shape DNA interpretation of Reuter et al. [23], in which the spectrum of the Laplace–Beltrami operator characterises the pocket beyond its local differential properties. We caution that this interpretation is hypothesis-generating and would benefit from joint analysis with explicit-water molecular dynamics on a curated subset of complexes. Bootstrap confidence intervals for each sub-block configuration on both the random and cluster-disjoint splits are reported in Figure A10 of Appendix B, and an alternative compact representation of the per-sub-block lift across split regimes is provided in Figure A11 of the same appendix.

### 2.5. External Benchmarks: CASF-2016 and LP-PDBBind DataSAIL S2

Table 3 compares our results against published scoring functions and graph neural networks on two external benchmarks. On CASF-2016 we observed a Pearson lift of +0.365 when the discrete differential geometry block was added to the concat baseline, taking the Pearson correlation from 0.253 to 0.618. The magnitude of this lift should be read in the context of an unusually low concat baseline at 0.253, which reflects the limited *k*-mer overlap between the CASF-2016 protein families and our PDBBind-Refined-derived training partition. The 3-mer baseline alone reaches only 0.248 on CASF-2016 (Table A3 of Appendix C). The CASF-2016 lift therefore documents the invariance of the geometric block under sequence-distribution shift between training and CASF-2016, rather than a 0.36 improvement over a competitive baseline. Concretely, our concat plus DDG model on CASF-2016 reaches a Pearson correlation of 0.618, on a par with the classical empirical scoring functions X-Score at 0.604 and AutoDock Vina at 0.604, while our DDG-only 59-dimensional model reaches 0.594. Modern atom-level graph neural network methods retain a clear advantage of approximately 0.22 in Pearson correlation on this benchmark, which we do not claim to match. Our finding is more modest and arguably more useful for practical pipelines, namely that an interpretable, architecture-light surface descriptor is by itself sufficient to recover classical scoring-function performance.

**Figure 7:**
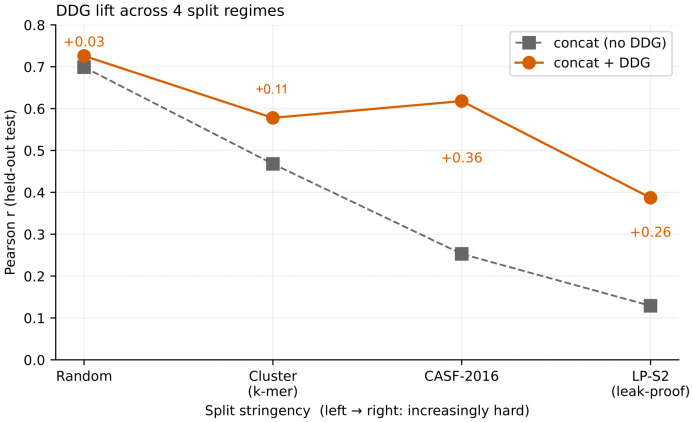
Pearson lift induced by the discrete differential geometry block across four split regimes of increasing stringency. The grey dashed line traces the fingerprint-and-sequence concat baseline, and the orange solid line traces the same baseline augmented with the 59-dimensional geometric block. Numerical labels report the per-regime Δr. As the train–test partition becomes stricter, the baseline collapses but the DDG-augmented model degrades more gently, so the lift grows from 0.03 on the random split to 0.36 on CASF-2016.

The same pattern holds on the strictest available leak-proof benchmark. On LP-PDBBind DataSAIL S2, where both ligands and proteins are disjoint between train and test, the concat baseline collapses to a Pearson correlation of 0.129 and the DDG-augmented variant rises to 0.387, a lift of +0.258. The DDG-only model reaches 0.456, again matching Pafnucy and AutoDock Vina, while trailing the IGN, GIGN and EHIGN family of atom-level networks by approximately 0.05. Across the four split regimes the lift ranges from +0.027 on the random split to +0.365 on CASF-2016 in a profile that grows monotonically with split stringency (Figure 7). The monotonic profile is the clearest evidence that the geometric content does not memorise training proteins; if it did, one would expect the lift to shrink under stricter partitioning rather than grow.

Because the practical purpose of a scoring function is to order candidate molecules rather than to reproduce their absolute affinities, we also evaluated the descriptor under the Spearman and Kendall rank correlations, which are the metrics used for the ranking power of the CASF benchmark. The rank-based coefficients follow the same qualitative pattern as the Pearson correlation and are reported in full in Table A3 and Table A4 of Appendix C. Adding the geometric block to the concat baseline lifts the Spearman correlation by +0.118 on cluster-disjoint testing, +0.361 on CASF-2016 and +0.246 on LP-PDBBind DataSAIL S2, and the Kendall correlation by +0.092, +0.253 and +0.172 respectively, so the conclusion that pocket-surface geometry contributes a leakage-robust signal does not depend on the choice of correlation coefficient. On CASF-2016 the DDG-only model reaches a Spearman correlation of 0.604 and a Kendall correlation of 0.425, and on the leak-proof partition, 0.444 and 0.310, mirroring its Pearson performance.

A direct comparison with RTMScore [34], a graph-transformer scoring function that learns a residue-atom distance-likelihood potential through a mixture density network, is informative but not a like-for-like contest on the scoring power metric. RTMScore attains state-of-the-art docking and screening power on CASF-2016, yet its developers report that its scoring and ranking power on the same benchmark fall well below the average because the training objective underuses experimental binding affinities. The descriptor studied here addresses the complementary regime, namely the recovery of a leakage-robust affinity signal in lightweight feature-based pipelines, so the two approaches are best seen as targeting different tasks within structure-based scoring rather than as competing on a single axis.

**Figure 8:**
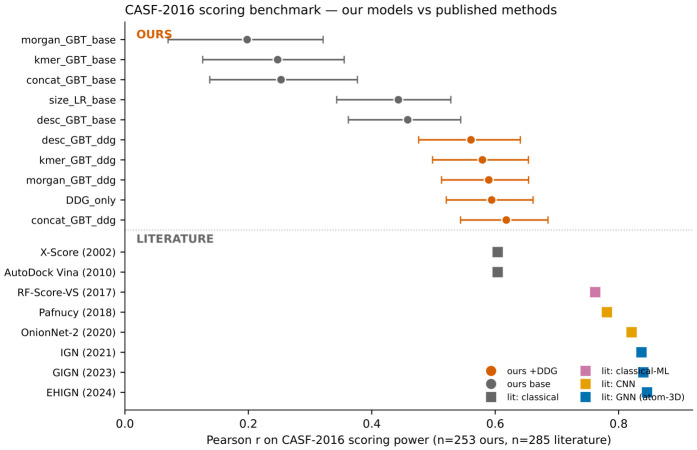
CASF-2016 scoring power benchmark. The top panel shows our results ($n_{\mathrm{test}}=253$ of 285), with orange circles for variants augmented with the DDG block and grey circles for the corresponding base models. Error bars are 95% bootstrap confidence intervals. The bottom panel shows published results on the same benchmark (full n=285), grouped by class: classical empirical scoring functions X-Score [5] and AutoDock Vina [4] (grey squares); the classical machine learning method RF-Score-VS (pink square); the convolutional networks Pafnucy [32] and OnionNet-2 [33] (yellow squares); and the atom-level three-dimensional graph neural networks IGN [8], GIGN [9] and EHIGN [10] (blue squares). The visual proximity of the GIGN and EHIGN markers reflects published Pearson values that differ by less than 0.01 on this benchmark; numerical values for all methods are tabulated in Table 3. Atom-level three-dimensional graph neural networks retain an advantage of approximately 0.22 in Pearson correlation over our feature-based concat plus DDG model.

**Figure 9:**
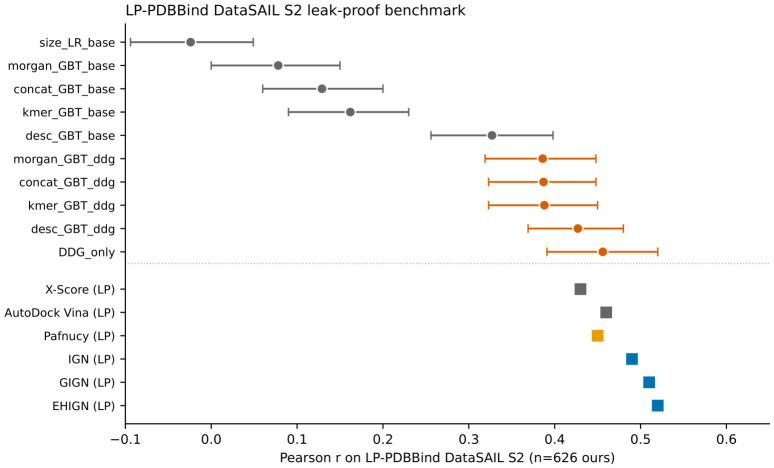
LP-PDBBind DataSAIL S2 leak-proof benchmark ($n_{\mathrm{test}}=626$ of 709), using the same conventions as Figure 8: grey circles for base configurations and orange circles for the same configurations augmented with the DDG block in the OURS panel, with 95% bootstrap confidence intervals; in the literature panel grey squares denote the classical empirical scoring functions X-Score [5] and AutoDock Vina [4], the yellow square denotes the convolutional network Pafnucy [32], and blue squares denote the atom-level three-dimensional graph neural networks IGN [8], GIGN [9] and EHIGN [10]. The DDG-only model at the top of the OURS panel reaches a Pearson correlation of 0.46, matching X-Score and AutoDock Vina, while atom-level three-dimensional graph neural networks lead by approximately 0.05.

A method-level comparison of our models against published scoring functions and graph neural networks on the CASF-2016 scoring power benchmark is shown in Figure 8, with the matched leak-proof comparison on LP-PDBBind DataSAIL S2 shown immediately afterwards in Figure 9.

### 2.6. SHAP Attribution Identifies ${\mathrm{HKS}}_{t_{0}}$ and $\lambda_{3}$ as Dominant Single Features

**Figure 10:**
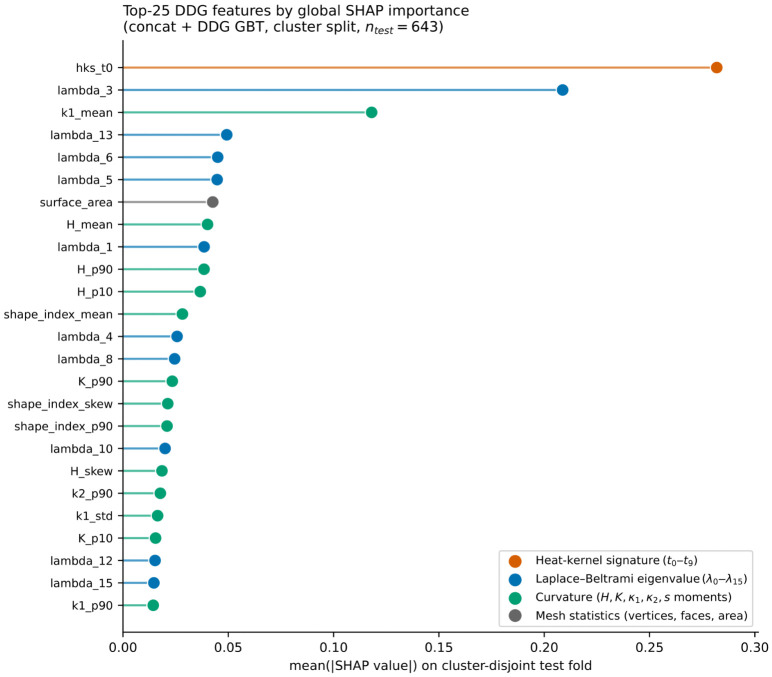
Top 25 discrete differential geometry features ranked by global SHAP importance on the cluster-disjoint test fold (n=643). The heat-kernel signature is colour-coded orange, Laplace–Beltrami eigenvalues blue, curvature moments green and mesh statistics grey.

**Figure 11:**
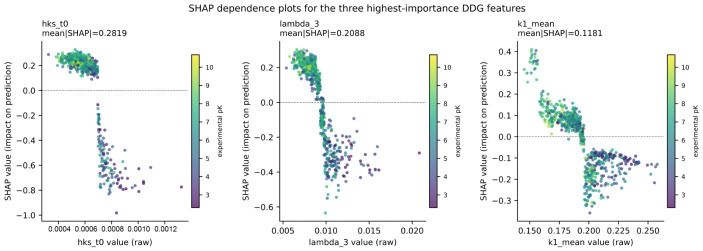
SHAP dependence plots for the three highest-importance discrete differential geometry features. Each point is one held-out test complex; colour encodes experimental pK. The non-monotonic structure of $\lambda_{3}$ in the middle panel suggests a bimodal contribution.

To localise which dimensions of the discrete differential geometry descriptor actually drive the lift, we computed TreeSHAP values [35] for the cluster-disjoint test fold of the concat plus DDG model (Figure 10). The three highest features by global mean absolute SHAP magnitude are the heat-kernel signature at the shortest diffusion time ${\mathrm{HKS}}_{t_{0}}$ at 0.28, the third Laplace–Beltrami eigenvalue $\lambda_{3}$ at 0.21, and the per-vertex mean of the maximum principal curvature $\langle \kappa_{1}\rangle$ at 0.12. Together these three account for more than half of the cumulative DDG SHAP magnitude. At the group level the sixteen Laplace–Beltrami eigenvalues collectively contribute a summed mean absolute SHAP of 0.53, followed by the ten heat-kernel signature time points at 0.30. After size normalisation to a per-feature mean, the heat-kernel signature and Laplace–Beltrami eigenvalues are essentially tied, at 0.030 and 0.033 respectively, and substantially above the per-feature curvature contributions of *H* at 0.025, $\kappa_{1}$ at 0.028, $\kappa_{2}$ at 0.011 and *K* at 0.011. The dependence plots in Figure 11 show a monotonically decreasing SHAP profile for ${\mathrm{HKS}}_{t_{0}}$ in the high-pK regime, consistent with a binding-affinity-discriminating role for the shortest-time-scale heat-kernel response, and a bimodal profile for $\lambda_{3}$ that suggests two distinct modes of pocket spectrum carrying complementary information about affinity.

### 2.7. Case Study of Best-Predicted and Worst-Predicted Complexes

**Figure 12:**
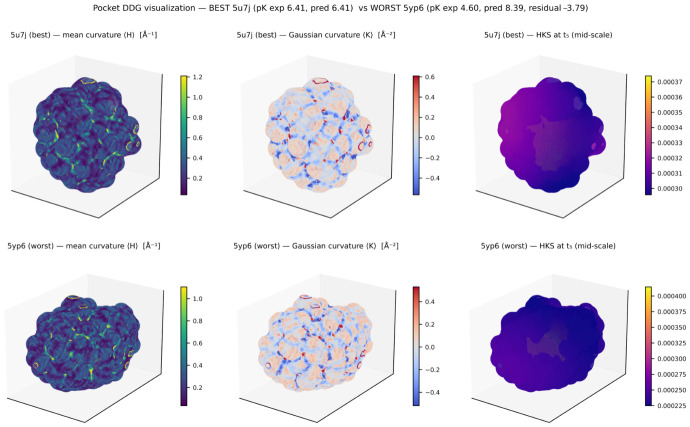
LA-SES surfaces of the two case-study complexes, coloured by per-vertex mean curvature *H*, Gaussian curvature *K*, and the heat-kernel signature at the mid-range diffusion time $t_{5}$. Black dots indicate ligand heavy-atom positions overlaid on the surfaces. The visibly broader heat-kernel signature distribution on the worst-predicted complex 5yp6 reflects an unusual spectral signature that the model is unable to discount.

To illustrate how the geometric content carried by the descriptor manifests at the level of individual binding pockets, we selected two contrasting cases from the cluster-disjoint test fold predictions of the concat plus DDG model, the smallest absolute residual and the largest absolute residual within the sequence-length range 100 to 500 and the pK range 4 to 10, restrictions intended to exclude peptide-like outliers. The best-predicted complex is the kinase-like target with PDB identifier 5u7j, where the model attains a residual of +0.001pK, and the worst-predicted complex is 5yp6, where the model overestimates affinity with a residual of −3.79pK. The LA-SES surfaces of the two complexes, coloured by per-vertex mean curvature, Gaussian curvature and the heat-kernel signature at the mid-range diffusion time $t_{5}$, are shown in Figure 12.

The per-ligand-atom geometric profiles, in which each column represents one ligand atom and each row represents one DDG feature, are provided as a within-ligand *z*-score heatmap in Figure A14 of Appendix D, and the per-vertex distributions of mean curvature, Gaussian curvature, shape index and HKS at $t_{5}$ across the two pockets are shown as kernel density estimates in Figure A15 of the same appendix. The visibly broader heat-kernel signature distribution on the worst-predicted complex, together with the unusually uniform per-atom geometric profile of its ligand, is consistent with the model’s overestimate, in which an ostensibly favourable pocket spectrum coexists with comparatively modest experimental affinity.

This case illustrates the principal practical failure mode of the descriptor. Because it characterises the pocket purely through its surface geometry and spectrum, the descriptor cannot, by construction, register the specific chemical determinants of affinity, such as the identity and protonation state of the contacting residues, directional hydrogen bonds, electrostatic complementarity or water-mediated bridges. When a pocket presents a geometrically favourable surface that is not matched by these chemical determinants, as in 5yp6, the descriptor tends to overestimate affinity. The descriptor is therefore best understood as a geometric prior on binding that is most reliable when geometric and chemical complementarity coincide and least reliable for pockets whose affinity is dominated by chemistry that the surface does not encode, a limitation that the hybrid mesh-to-atom architectures discussed in Section 3 are intended to address by letting atomic chemical features attend directly to the surface.

### 2.8. Robustness Sensitivity to Cluster Threshold and Auxiliary Splits

To verify that the lift attributed to the discrete differential geometry descriptor is not an artefact of a particular Jaccard threshold or partitioning convention, we repeated the analysis at three thresholds, t=0.3, t=0.4 and t=0.5, and on a time-like alphabetical split obtained by sorting complexes by PDB identifier and assigning the alphabetically last 20% to the test partition. The Pearson lift increases monotonically as the threshold drops and the cluster-disjointness becomes stricter, with Δr values of +0.129 at t=0.3, +0.111 at t=0.4 and +0.076 at t=0.5, as plotted in Figure A16 of Appendix E. The time-like alphabetical split yields a positive lift of +0.068, smaller than the cluster-disjoint values but consistent with a leakage-robust geometric signal. A complete robustness summary, including the five-seed ensemble means and standard deviations, the three Jaccard threshold values, the permutation test *p*-value, the time-like alphabetical lift and the two external benchmark lifts, is provided in Table A5 of the same appendix.

### 2.9. Complementary Architectural Analysis: DDG Injection in a Distance-Based Graph Neural Network

To delineate the operational scope of pocket-surface discrete differential geometry as a feature class, we tested four mechanistic strategies for injecting the same descriptor into a SchNet-style distance-based graph neural network with four InteractionBlock layers, 96 hidden channels, a 6 Å spatial cutoff and 32 Gaussian radial basis functions, all under an identical training protocol. The first strategy concatenates the 59-dimensional descriptor with the pooled graph embedding before the readout multilayer perceptron (B.2). The second strategy projects the per-atom 16-dimensional geometric slice through a SiLU multilayer perceptron and adds it to the atomic embedding before message passing (B.3.1). The third strategy applies a DDG-conditional cross-attention layer in which ligand atoms attend to pocket atoms with attention scores biased by the cosine similarity of per-atom geometric vectors as in Equation (4) (B.3.2). The fourth strategy augments the standard distance-based edge features with the three additional Gaussian-expanded geometric channels of Equation (5) in the spirit of CurvAGN (B.3.3).

Table 4 reports the resulting Pearson correlations and their differences from the matched atom-only baselines retrained alongside each variant. On the cluster-disjoint test fold, all four mechanisms produce Δr values that fail to reach the 0.02 marginality threshold, namely −0.004 for B.2, +0.011 for B.3.1 with a 95% bootstrap confidence interval of [−0.010,+0.033], +0.016 for B.3.2 and −0.006 for B.3.3. The mean cluster Δr across all four mechanisms is +0.004, statistically indistinguishable from zero. A 2000-iteration paired-difference bootstrap on the B.3.1 versus base comparison gives p(Δr>0)=0.84, below the conventional 0.95 acceptance level. On the random split the picture is more variable. B.3.1 yields the largest lift at +0.018, while B.3.2 produces a regression of −0.021 relative to its own atom-only baseline and B.3.3 produces a regression of −0.006. The mean random Δr across the four mechanisms is −0.001, again indistinguishable from zero. The negative lifts observed in B.3.2 and B.3.3 indicate that the additional architectural complexity, namely the parameters introduced by cross-attention and the 48 extra edge dimensions introduced by curvature-conditioned edges, contributes variance that can exceed the geometric signal in this small-backbone setting. The complete per-mechanism best-epoch results, including the matched atom-only baselines retrained jointly within each variant, are reported in Table A6 of Appendix F.

The four-mechanism consistency, across mechanistically distinct injection points covering readout-level, embedding-level, post-message-passing attention and edge-feature modulation, supports a focused interpretation. Within this SchNet-style distance-based backbone, the explicit injection of discrete differential geometry information does not produce a statistically significant performance gain on cluster-disjoint testing. The architectural variation we examined is broad along the injection-mechanism axis but narrow along the backbone-family axis, and our SchNet baseline at a Pearson correlation of approximately 0.68 on the random split lies below published large-scale three-dimensional atom-level graph neural networks at approximately 0.84 on CASF-2016. Read together with the strong feature-based effect documented above, this absence of lift is best interpreted not as evidence that the geometric content is uninformative but as evidence that distance-based message passing on atomic coordinates already captures much of it. The result is informative for pipeline design rather than detrimental to the descriptor itself, and it identifies a clear architectural frontier, namely the direct attention of atomic representations to mesh-level geometry, that we discuss in the next section.

## 3. Discussion

Our analyses position pocket-surface discrete differential geometry as a leakage-robust feature class for protein–ligand binding affinity prediction with a clearly delineated operational scope. In gradient-boosted tree pipelines that operate on fixed-size feature vectors and lack direct access to three-dimensional atomic coordinates, the 59-dimensional descriptor delivers consistent and statistically significant gains across progressively stricter evaluation regimes. The Pearson lift grows monotonically with split stringency, from 0.027 on a random partition to 0.111 on a *k*-mer Jaccard cluster-disjoint partition, to 0.258 on the leak-proof LP-PDBBind DataSAIL S2 partition and to 0.365 on the CASF-2016 core set, in a profile that confirms the geometric content captures genuine binding determinants rather than memorisation artefacts. The descriptor used in isolation reaches Pearson correlations between 0.456 and 0.594 on external benchmarks, on a par with the long-standing classical scoring functions X-Score and AutoDock Vina. The ablation, the permutation test, the five-seed ensemble, the surface area partial-out control and the TreeSHAP analysis collectively identify the heat-kernel signature, particularly at short and mid-range diffusion times, as the dominant carrier of the signal. These spectral features encode multi-scale information about the pocket, including local curvature, cavity dimensions relevant to desolvation, and the global enclosure and rigidity modes that influence the entropic component of binding.

The complementary architectural analysis sharpens rather than contradicts this picture. The same descriptor injected into a SchNet-style distance-based graph neural network through four mechanistically distinct strategies, namely global concatenation at the readout, per-atom projection at the embedding stage, DDG-conditional cross-attention biased by per-atom geometric similarity, and curvature-conditioned edge features that augment distance-based edge attributes with differences of mean and principal curvature and with heat-kernel cosine distance, produces Δr values in the range [−0.021,+0.018] across both splits, never exceeds the 0.02 marginality threshold on cluster-disjoint testing and has a mean of approximately 0.004. The four-fold consistency, across node-level, attention-level and edge-level injection points, is consistent with the interpretation that distance-based message passing on atomic coordinates within this small backbone already encodes a substantial part of the geometric information that the discrete differential geometry block makes explicit. We emphasise, however, that redundancy of geometric information is not the only possible explanation for the absence of a lift. The result may equally reflect the limited capacity and short training schedule of the deliberately compact backbone, the specific fusion mechanisms we tested, rather than the full space of possible ones, or limited statistical power, given that multi-seed confidence intervals on the graph neural network experiments were not collected. The most defensible reading is therefore the narrower one, namely that under the present SchNet-style backbone and the four tested integration strategies, no statistically significant improvement was observed, and that the residual signal does not justify the added architectural complexity in this specific setting. We emphasise that our SchNet baseline at a Pearson correlation of approximately 0.68 on the random split is small and short-trained relative to published state-of-the-art three-dimensional graph neural networks at approximately 0.84 on CASF-2016, so this conclusion is best stated as architecture dependence within the SchNet family rather than as a universal claim about all three-dimensional message-passing networks. The result is most useful read as a pipeline-design finding rather than as a competitive benchmark on large 3D networks, and it identifies a concrete research direction in the form of hybrid mesh-to-atom attention that we discuss below.

These findings carry direct practical implications for computational drug design. For resource-constrained or early-stage virtual screening pipelines that rely on sequence or ligand-fingerprint features alone, or where full three-dimensional docking and equivariant message passing are prohibitively expensive at the required throughput, the discrete differential geometry block offers a lightweight, interpretable and computationally efficient complement that recovers classical scoring-function performance from a single 59-dimensional surface descriptor. The descriptor adds an information channel that is invariant under sequence distribution shift, as documented by the CASF-2016 lift, and it does so at a cost dominated by mesh construction and Laplace–Beltrami eigendecomposition. To quantify this claim, the full discrete differential geometry batch over the 3285 complexes completed in approximately 36 min on a single NVIDIA T4 graphics processing unit, an average of roughly 0.65 s per complex, including implicit-surface sampling, marching-cubes meshing, Taubin smoothing, quadric-error decimation and the spectral computation, after which each feature-based fit, evaluate and bootstrap cycle required at most five minutes. This descriptor-generation cost is incurred once per complex and is modest relative to the training of large three-dimensional graph neural networks, which typically require many graphics-processing-unit hours; the lightweight characterisation therefore refers to the end-to-end cost of computing and using the descriptor rather than to its dimensionality alone. The interpretability is a non-trivial advantage in medicinal chemistry contexts where post hoc rationalisation of model predictions is required for internal review or regulatory submission. The TreeSHAP attributions identify the short-time-scale heat-kernel signature and the low-order Laplace–Beltrami eigenvalues as the dominant features, and both correspond to chemically meaningful properties of the binding pocket, namely local curvature and global enclosure, that medicinal chemists can inspect in the context of structural hypotheses about hot-spots, druggability and selectivity.

For modern three-dimensional graph neural network pipelines, our results suggest that the explicit injection of pocket-surface discrete differential geometry is largely redundant under current distance-based message-passing paradigms, at least within the SchNet family we tested. The natural next direction is the development of hybrid mesh-to-atom architectures, in which ligand atoms attend directly to the full pocket surface rather than to per-atom projections of mesh quantities, weighted by curvature or spectral signatures. Such an architecture would allow the model to dynamically select the geometric modes most relevant to a given ligand-pocket pair, would treat the surface as a first-class object amenable to multi-scale spectral analysis, and would, in principle, preserve the rotational and translational invariance that motivates the use of discrete differential geometry in the first place. The architectural design space this opens up is broad, including, for example, surface attention layers conditioned on heat-kernel signature similarity at multiple diffusion times, or equivariant transformers that operate jointly on atomic point clouds and on mesh patches sampled around the binding cleft.

We acknowledge several limitations of the present study. Our experiments were conducted on PDBBind v2020 with partial mirror coverage, namely 3285 of 3558 refined-set complexes, 253 of 285 CASF-2016 complexes and 626 of 709 LP-PDBBind DataSAIL S2 complexes, with the missing complexes itemised in Appendix A. The complementary architectural analysis used a deliberately compact SchNet-style backbone chosen to enable a controlled feature-injection ablation under a constrained compute budget, and our SchNet baseline at approximately 0.68 on the random split lies below published large-scale models at approximately 0.84 on CASF-2016, so the architectural conclusion is restricted to the SchNet family. Multi-seed confidence intervals on the graph neural network results were not collected due to compute budget, and the reported Δr values are best-epoch single-seed differences whose uncertainty is bounded only by the four-mechanism consistency we report. The architectural space we examined does not include equivariant networks such as E(3)-NN or NequIP, large-scale distance-based networks at IGN or GIGN scale, or architectures that operate directly on the LA-SES mesh rather than projecting per-atom geometric quantities to atoms. Whether the discrete differential geometry block provides additive value in those architectures remains an open and high-priority question that we identify for future work.

A related limitation concerns the scope of the baseline comparison. Our external comparison in Table 3 positions the descriptor against classical empirical scoring functions and against published atom-level three-dimensional graph neural networks, but it does not include a like-for-like re-evaluation of stronger modern architectures under the identical leakage-protected protocol, in particular, equivariant networks, large interaction-focused protein–ligand networks, surface-based geometric deep learning models such as MaSIF and dMaSIF, and the persistent-homology, persistent-Laplacian and Mayer-homology topological descriptors that reach state-of-the-art accuracy on standard benchmarks. The literature values we cite were obtained on the full benchmarks rather than on our exact partitions, so they bound the competitive gap only approximately and do not control for leakage in the same way as our internal protocol. Our results therefore establish that the descriptor is a competitive lightweight feature-engineering approach, but they do not establish whether it offers complementary value beyond these stronger three-dimensional and surface-based models. Settling this question would require retraining each such model under the DataSAIL S2 and cluster-disjoint protocols used here, which we regard as an important direction for future work.

The exclusion of the 273 complexes for which a structure could not be retrieved in a parseable form or for which mesh computation failed may introduce a selection effect. The large majority of these exclusions arises from the structure being absent from the public mirror, a cause that is unrelated to pocket geometry or to binding affinity and is therefore expected to act approximately at random, while a smaller fraction reflects rejection by RDKit at the strictest sanitisation level, which may correlate weakly with unusual ligand chemistry. The retained subset spans the same pK dynamic range and distribution as the full labelled set (Figure 2), which argues against a strong affinity-dependent bias, but we cannot fully exclude a residual selection effect, and confirmation on a complete-coverage structure source would remove this caveat.

## 4. Materials and Methods

This section describes each stage of the analysis workflow of Figure 1 in detail, beginning with the construction of the ligand-aware pocket surface and the extraction of its geometric descriptors, continuing with the two parallel modelling pipelines we evaluated, and concluding with the leakage-protected splits and external benchmarks used for assessment.

### 4.1. Datasets and Affinity Labels

PDBBind v2020 protein–ligand structures and their associated binding affinities, expressed throughout this work as $\mathrm{pK}=-\log_{10}K_{\left[M\right]}$ from $K_{d}$ or $K_{i}$ values, were obtained from the public Hugging Face mirror jglaser/pdbbind_complexes containing 16,079 complexes, joined with the BindingDB-derived affinity table jglaser/binding_affinity of 1,836,729 records on the composite key formed by the protein sequence and the canonical ligand SMILES. After filtering for sequence length between 20 and 2000, pK∈[2,11] and uniqueness of PDB identifier, we retained 3558 labelled complexes. Three-dimensional protein structures and ligand SDF files were retrieved from the THU-ATOM/PDBbind mirror, of which 3285 complexes (92.3%) were available in parseable form, the remaining 273 were either absent from the mirror or rejected by RDKit at the strictest sanitisation level. Affinity labels for the CASF-2016 core set and the DataSAIL-curated LP-PDBBind splits were obtained from Zenodo records 8091220 and 17376012, respectively. Structure files for CASF-2016 and the LP-PDBBind DataSAIL S2 test partition were retrieved through the same mirror channel with 88.8% and 88.3% coverage, yielding $n_{\mathrm{test}}=253$ for CASF-2016 and $n_{\mathrm{test}}=626$ for LP-PDBBind DataSAIL S2. The complete filtration log from the raw mirrors to the final analysis set, and an itemised list of the complexes absent from the mirror, are reported in Table A1 of Appendix A.

### 4.2. Ligand-Aware Solvent-Excluded Surface

For every complex we constructed a ligand-aware solvent-excluded surface (LA-SES) using a probe of radius 1.4 Å rolled over protein heavy atoms within 10 Å of any ligand heavy atom. The implicit signed distance field was sampled on a grid of resolution 0.8 Å, or 1.12 Å for very large bounding boxes, and converted to a triangle mesh by marching cubes. The largest connected component was retained, smoothed with eight Taubin iterations (λ=0.5, μ=−0.53), and decimated by quadric-error-metric edge collapse to at most 12,000 faces. The resulting mesh sizes were uniform across the dataset, with $n_{\mathrm{vertices}}=6000\pm 200$ and $n_{\mathrm{faces}}=12,000\pm 100$.

### 4.3. Discrete Differential Geometry Descriptors

**Figure A4:**
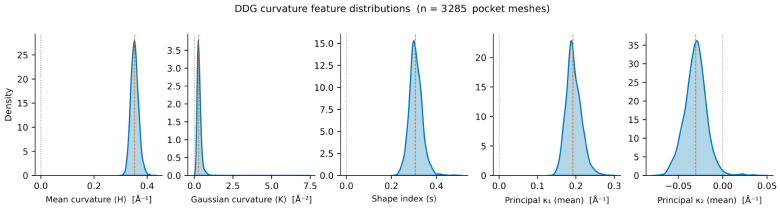
Per-vertex DDG curvature distributions across the 3285 pocket meshes, summarised as kernel density estimates of the per-vertex mean for each of mean curvature 〈H〉 in ${Å}^{-1}$, Gaussian curvature 〈K〉 in ${Å}^{-2}$, shape index 〈s〉, and principal curvatures $\langle \kappa_{1}\rangle$ and $\langle \kappa_{2}\rangle$. The vertical orange dashed lines mark the dataset-wide medians of each distribution, and the vertical grey dotted lines mark zero on the horizontal axis as a reference for the sign of the curvature.

**Figure A5:**
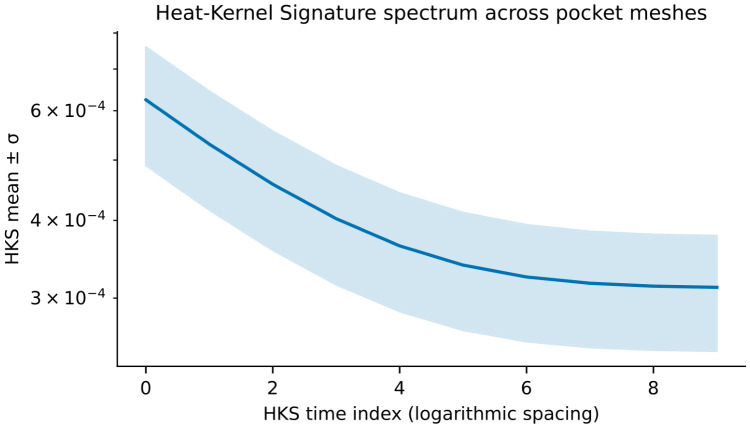
Heat-kernel signature spectrum across the 3285 pocket meshes. The solid line traces the dataset-wide mean and the shaded band shows ±σ. The monotone decrease with logarithmically spaced *t* reflects the diffusion-time dependence of the heat kernel and provides the multi-scale shape spectrum on which the per-vertex feature vector is sampled.

**Figure A6:**
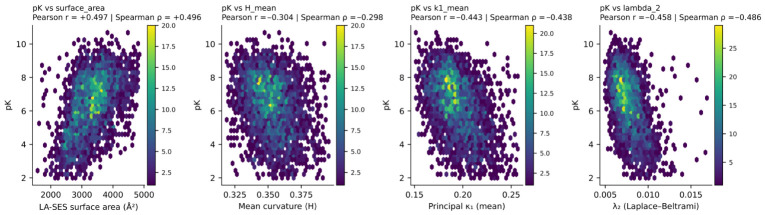
Hexbin density plots of pK against the four most informative individual DDG channels: LA-SES surface area, mean curvature 〈H〉, principal $\langle \kappa_{1}\rangle$ and the second Laplace–Beltrami eigenvalue $\lambda_{2}$. Pearson and Spearman coefficients on the full set (n=3285) are shown above each panel. The surface area channel carries the strongest single-feature rank correlation with pK at approximately −0.486 in Spearman terms, while individual curvature and Laplace–Beltrami channels have weaker per-channel correlations. The leakage-robust signal that drives the lift values reported in the main text arises from the joint distribution of the 59-dimensional descriptor rather than from any single feature.

**Figure A7:**
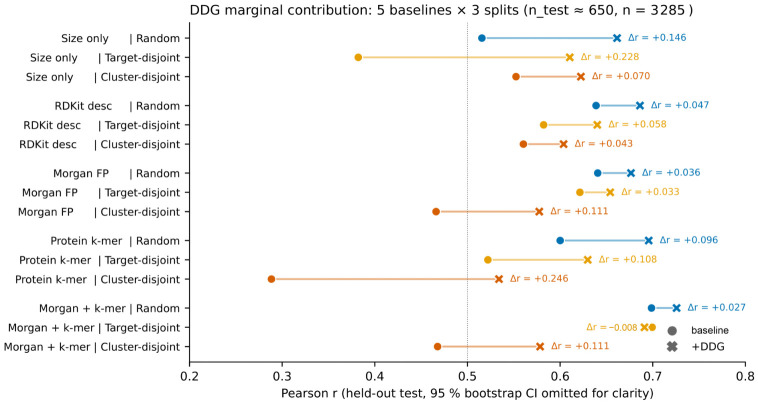
Marginal contribution of the discrete differential geometry block to each baseline feature set, across the three internal split regimes. Grey circles correspond to the baseline alone and grey crosses to the same baseline augmented with the geometric block, with the per-pair Pearson lift labelled. The strongest absolute lift is observed on the protein 3-mer baseline under the cluster-disjoint split, at +0.246, reflecting that the geometric features successfully replace the memorisation signal that the cluster split removes.

**Figure A8:**
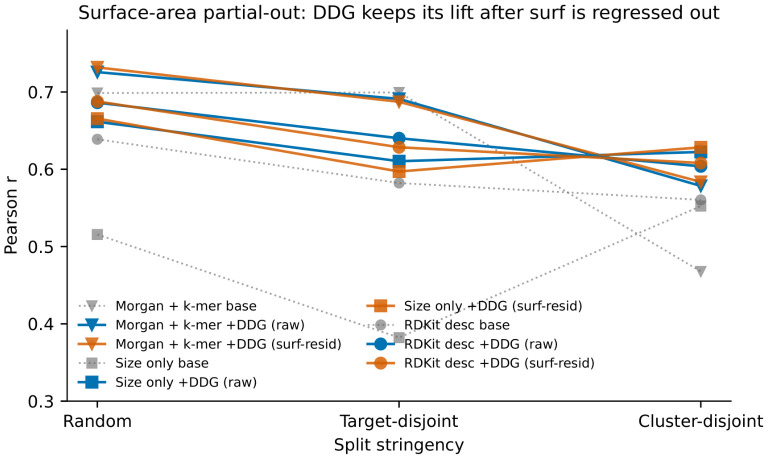
Surface-area partial-out control. The 59-dimensional discrete differential geometry block was first residualised against the surface area channel by per-channel linear regression and then concatenated to each baseline. The lift over the base model survives this control and is comparable to the raw lift, indicating that the signal is not an artefact of pocket size correlating with ligand size.

**Figure A9:**
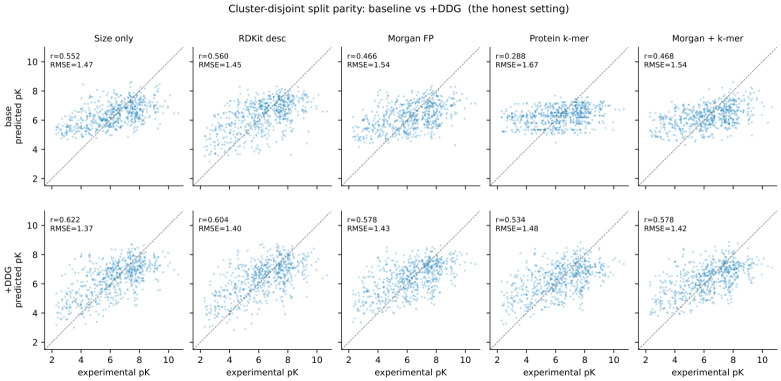
Parity plots for base versus augmented models on the cluster-disjoint test fold (t=0.4, n=643) across the five baseline feature sets. The top row shows the base configurations and the bottom row the same configurations augmented with the discrete differential geometry block. The augmentation increases the Pearson correlation and visibly compresses the residual scatter, particularly in the high-pK tail.

**Figure A10:**
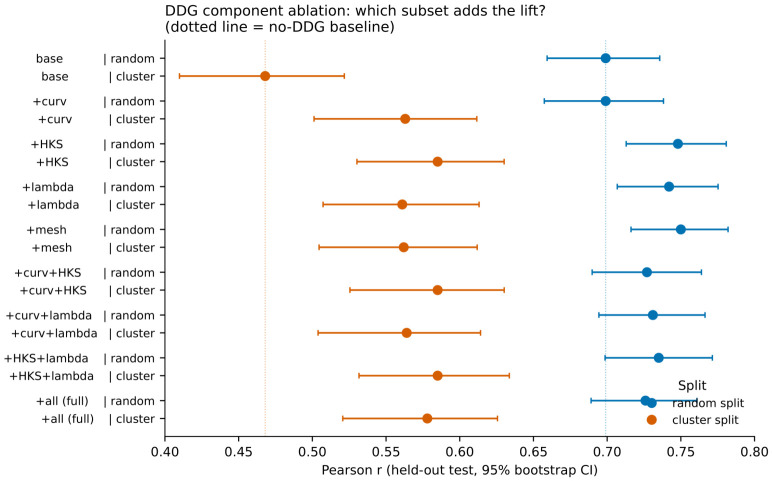
Per-component DDG ablation. Each row corresponds to one feature configuration evaluated on either the random split (blue) or the cluster-disjoint split (orange), with 95% bootstrap confidence intervals. Vertical dotted lines mark the no-DDG baseline. The ten-feature heat-kernel signature alone matches the full 59-dimensional block, and combinations of sub-blocks are sub-additive.

**Figure A11:**
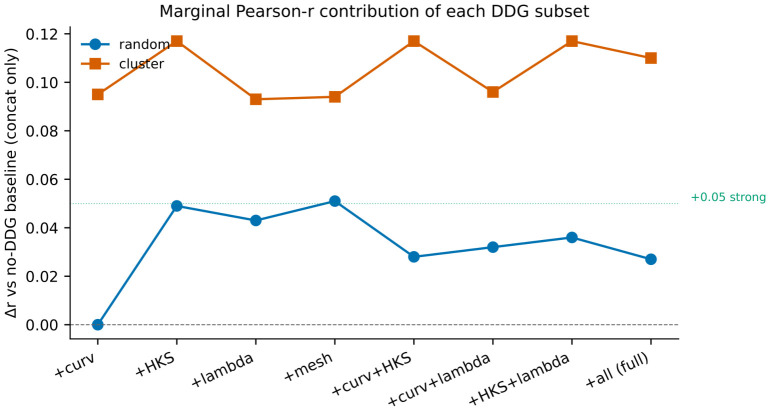
Pearson lift per discrete differential geometry sub-block plotted as a curve, illustrating the dominance of the heat-kernel signature in a more compact representation than that of Figure A10. The horizontal dotted line marks the 0.05 meaningful threshold; the cluster split lift exceeds this threshold for every sub-block.

**Figure A14:**
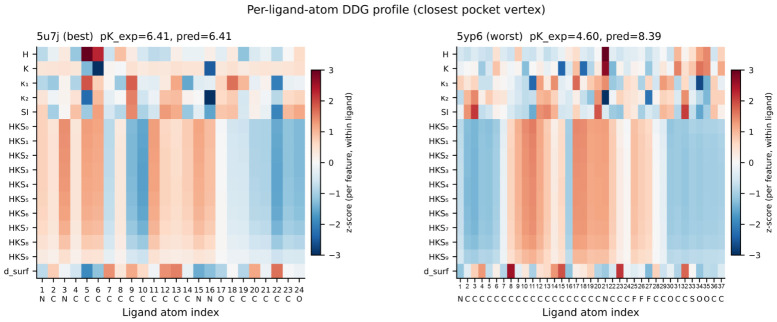
Per-ligand-atom DDG profile. Each column represents one ligand atom, with the element symbol below; each row is one DDG feature and the colour encodes the within-ligand *z*-score, with red representing high relative values and blue representing low relative values. The 5yp6 ligand exhibits a more uniform pattern, suggesting that its larger set of atoms each sees a similar pocket environment despite the modest experimental affinity.

**Figure A15:**
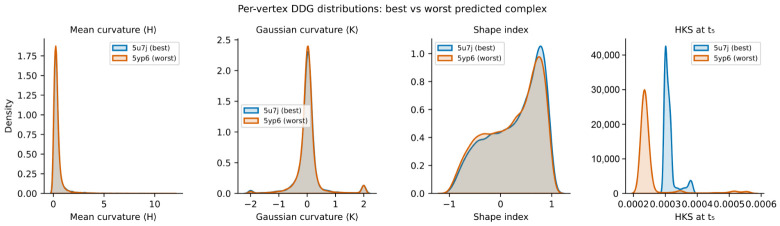
Kernel density estimates of per-vertex DDG distributions for the two case-study complexes. The mean and Gaussian curvature distributions are similar between the two pockets, while the heat-kernel signature at $t_{5}$ panel reveals a markedly different spectral signature, consistent with the model’s overestimate on 5yp6.

**Figure A16:**
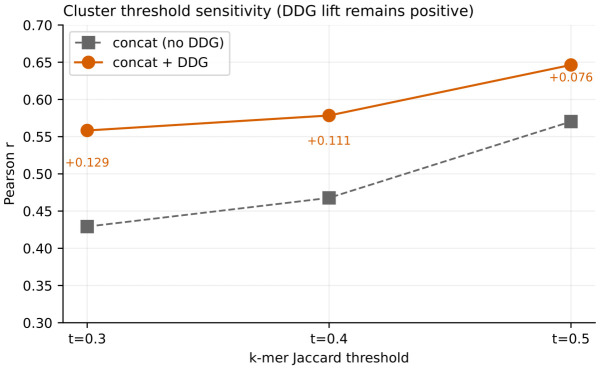
Cluster threshold sensitivity. Three Jaccard thresholds were tested, t=0.3 (stricter), t=0.4 (default) and t=0.5 (looser). The Pearson lift increases monotonically as the threshold drops, that is as cluster-disjointness becomes more stringent. This pattern is consistent with the role of pocket-surface discrete differential geometry as a leakage-robust signal that becomes proportionally more valuable when alternative memorisation channels are foreclosed.

On every LA-SES mesh we computed five families of per-vertex geometric quantities, summarised by their distributional moments to yield a fixed-size descriptor. The mean curvature was obtained via the cotangent Laplacian as

(1) $$H=\frac{1}{2}\;∥\Delta_{M}p∥,$$

where $\Delta_{M}$ is the discrete Laplace–Beltrami operator on the mesh and p denotes the embedding of a vertex in three-dimensional space. The Gaussian curvature *K* was computed from the angle deficit divided by the Voronoi mixed area, and the principal curvatures $\kappa_{1},\kappa_{2}$ were extracted using libigl’s principal_curvature routine. From the principal curvatures we derived the shape index

(2) $$s=\frac{2}{\pi }\;\arctan\;\left(\frac{\kappa_{1}+\kappa_{2}}{\kappa_{1}-\kappa_{2}}\right),$$

which maps the local surface geometry to a scalar value in [−1,1] that distinguishes cup, rut, saddle, ridge and cap configurations. The heat-kernel signatures were evaluated at ten logarithmically spaced diffusion times *t* as

(3) $${\mathrm{HKS}}_{t}\left(v\right)\;=\;\sum_{i=1}^{16}e^{-\lambda_{i}t}\;\phi_{i}{\left(v\right)}^{2},$$

where $\lambda_{i}$ and $\phi_{i}$ are the *i*-th eigenvalue and eigenfunction of the discrete Laplace–Beltrami operator. Each per-vertex distribution was summarised by six moments, namely the mean, standard deviation, the tenth and ninetieth percentiles, skewness and kurtosis. To these moment summaries we appended the leading sixteen Laplace–Beltrami eigenvalues and three mesh statistics, namely the number of vertices, the number of faces and the surface area. The concatenation produced a fixed 59-dimensional per-complex descriptor. The dataset-wide kernel density estimates of the per-vertex curvature features and of the heat-kernel signature spectrum across all 3285 pocket meshes are provided in Figure A4 and Figure A5 of Appendix B. Per-channel rank correlations between individual DDG features and pK on the full set, including LA-SES surface area, mean curvature 〈H〉, principal $\langle \kappa_{1}\rangle$ and the second Laplace–Beltrami eigenvalue $\lambda_{2}$, are summarised in Figure A6 of the same appendix.

### 4.4. Auxiliary Feature Blocks

For comparative analysis and as baselines, we computed four conventional feature blocks. The first is a one-dimensional ligand size baseline equal to the heavy-atom count of the ligand. The second is a nine-dimensional vector of canonical RDKit descriptors, namely MolWt, HeavyAtoms, RotBonds, LogP, TPSA, HBA, HBD, Rings and AromaticRings. The third is a 1024-bit Morgan circular fingerprint of radius 2. The fourth is a top-1024 protein 3-mer count vector obtained by applying DictVectorizer to all length-three substrings of the protein sequence and selecting the most frequent features by document frequency. The combined fingerprint and 3-mer baseline, referred to throughout as concat, has 2048 dimensions, and the addition of the discrete differential geometry block produces a 2107-dimensional feature vector.

### 4.5. Train–Test Splits

We evaluated four split regimes on the labelled set of 3285 complexes. The random regime used an 80/20 train–test partition with seed 42. The target-disjoint regime forbade any full sequence shared between train and test partitions, with seed 43. The *k*-mer Jaccard cluster-disjoint regime first clustered unique sequences by greedy single-linkage on k=3 amino-acid *k*-mer Jaccard similarity at thresholds t∈{0.3,0.4,0.5}, and then constructed train and test partitions whose cluster assignments were disjoint, with seed 44. The time-like alphabetical regime sorted complexes by PDB identifier and assigned the alphabetically last 20% to the test partition. For external benchmarking we used the CASF-2016 core set of 285 complexes as defined by Su et al. [6] and the leak-proof LP-PDBBind DataSAIL S2 partition of 709 complexes as distributed by the DataSAIL benchmark [20]. For both external benchmarks all overlapping PDB identifiers were removed from the training partition, yielding $n_{\mathrm{train}}$ between 3212 and 3215 depending on the benchmark.

### 4.6. Feature-Based Models

All feature-based models were trained with HistGradientBoostingRegressor from scikit-learn 1.4 using a Huber loss, a learning rate of 0.05, a maximum tree depth of six and no early stopping, with $n_{\mathrm{iter}}=300$ for feature counts at or below 100 and $n_{\mathrm{iter}}=200$ for higher feature counts. Linear regression was used for the size-only baseline.

### 4.7. SchNet-Style Atom-Level Graph Neural Network and DDG Injection Mechanisms

For the complementary architectural analysis, we employed a SchNet-style distance-based graph neural network with four InteractionBlock layers, 96 hidden channels, a 6 Å spatial cutoff, 32 Gaussian radial basis functions, separate ligand and pocket atom-type embeddings, a ligand-restricted global sum-pool and a three-layer multilayer perceptron readout. Training used a Smooth-L1 loss, the AdamW optimiser with $\beta_{1}=0.9$, $\beta_{2}=0.999$ and weight decay $10^{-5}$, an initial learning rate of $2\times 10^{-4}$ with cosine decay to $10^{-5}$, gradient clipping at norm 5, batch size 8, between 30 and 35 epochs and deterministic seed 42. The backbone was deliberately kept compact to enable a controlled feature-injection ablation under a constrained compute budget, and we therefore stress that the SchNet baseline reported here, which reaches a Pearson correlation of approximately 0.68 on the random split, lies below the published state-of-the-art for atom-level graph neural networks at approximately 0.84 on CASF-2016. Our architectural conclusions are accordingly framed as feature-injection sensitivity within the SchNet family rather than as a generalisation to all three-dimensional atom-level networks.

The first injection mechanism, denoted B.2, performs global concatenation of the 59-dimensional per-complex DDG descriptor with the pooled graph embedding immediately before the multilayer perceptron readout. No information is shared between the geometric descriptor and the per-atom message passing. The second mechanism, B.3.1, performs per-atom projection of geometric information at the embedding stage. For each ligand and pocket atom, the nearest LA-SES mesh vertex is identified by Euclidean distance and a 16-dimensional per-atom geometric slice is gathered, comprising the per-vertex mean and Gaussian curvatures, the principal curvatures, the shape index and the ten-dimensional heat-kernel signature at that vertex. This slice is projected through a two-layer SiLU multilayer perceptron of sizes 16→96→96 and added to the atomic embedding before the first message-passing block; the remainder of the backbone is unchanged. The third mechanism, B.3.2, applies a DDG-conditional cross-attention layer after the four message-passing blocks, in which ligand atom embeddings serve as queries and pocket atom embeddings serve as keys and values within a four-head attention layer of width 96. The pre-softmax attention score between query atom *i* and key atom *j* is biased by an additive geometric term according to

(4) $${\tilde{\alpha }}_{ij}\;=\;\alpha_{ij}+\beta \;\cos\;\left({\mathrm{DDG}}_{i},\;{\mathrm{DDG}}_{j}\right),$$

where $\alpha_{ij}$ is the standard scaled dot-product attention score, ${\mathrm{DDG}}_{i}$ and ${\mathrm{DDG}}_{j}$ are the same 16-dimensional per-atom geometric slices used in B.3.1, cos denotes the cosine similarity and β=1 is a fixed scalar. The attention output is added back to the ligand embeddings as a residual, ligand atoms are sum-pooled and the readout multilayer perceptron is applied. The fourth mechanism, B.3.3, implements a curvature-conditioned edge-feature scheme in the spirit of CurvAGN. Standard 32-dimensional Gaussian-expanded distances are augmented with three additional geometric channels, each Gaussian-expanded over sixteen centres,

(5) $$\Delta H_{ij}=|H_{i}-H_{j}|,\;{\Delta \kappa }_{ij}=|\kappa_{1,i}-\kappa_{1,j}|,\;d_{ij}^{\mathrm{HKS}}=1-\cos\;\left({\mathrm{HKS}}_{i},{\mathrm{HKS}}_{j}\right),$$

where the per-vertex quantities at atomic positions are evaluated at the corresponding nearest mesh vertices. The first InteractionBlock edge multilayer perceptron is widened to ingest the resulting 80-dimensional edge attribute, while downstream blocks are unchanged. All four mechanisms share the same backbone, training schedule, hyperparameters and per-split atom-only baselines retrained jointly within the same compute session to control for stochastic seed effects. Multi-seed confidence intervals on the GNN results were not collected due to compute budget, and the reported Pearson lifts should accordingly be read as best-epoch single-seed point estimates whose reliability is bounded by the four-fold consistency across mechanistically distinct injection points reported in the Results.

### 4.8. Statistical Evaluation

We report the Pearson correlation alongside the Spearman and Kendall rank correlations, together with root-mean-square error and mean absolute error, with 95% confidence intervals derived from 500 bootstrap resamples of the test fold. The two rank correlations are reported because the practical use of a scoring function in virtual screening is the correct ordering of candidate molecules, for which rank-based measures are more directly informative than the value-sensitive Pearson coefficient. Pairwise significance of the lift induced by adding the discrete differential geometry block was assessed by a 5000-iteration paired permutation test, in which for each held-out sample the model assignment, base or with DDG, was randomly swapped, and the resulting null distribution of the Pearson difference Δr was compared against the observed Δr. Robustness was further evaluated through a five-seed ensemble that varied both the cluster-split random number generator seed across 44 to 48 and the gradient-booster seed across 0 to 4.

### 4.9. Reproducibility

The feature-based experiments were conducted on a single Apple Silicon laptop, while the discrete differential geometry batch processing and the graph neural network training were carried out on Google Colaboratory with an NVIDIA T4 GPU. End-to-end runtime was approximately 36 min for the 3285-complex DDG batch, less than five minutes per feature-based configuration, and approximately two minutes per 30-epoch graph neural network seed. To make the pipeline reproducible without reference to the released code, the mesh-generation parameters, the gradient-boosted tree hyperparameters and the exact random seeds used for every split and ensemble are consolidated in Table A8, and the complete list of software versions used in the analysis pipeline is provided in Table A7, both in Appendix G.

## 5. Conclusions

We have presented a controlled, leakage-protected and architecturally explicit evaluation of pocket-surface discrete differential geometry as a feature class for protein–ligand binding affinity prediction. The 59-dimensional descriptor, computed from the ligand-aware solvent-excluded surface and combining mean, Gaussian and principal curvature distributions with the leading sixteen Laplace–Beltrami eigenvalues and a ten-point heat-kernel signature, delivers consistent and statistically significant performance gains in gradient-boosted tree pipelines across progressively stricter evaluation regimes, including a Pearson correlation lift of 0.111 on a *k*-mer Jaccard cluster-disjoint split with permutation $p<2\times 10^{-4}$, 0.258 on the leak-proof LP-PDBBind DataSAIL S2 partition and 0.365 on CASF-2016. The descriptor used in isolation reaches Pearson correlations between 0.456 and 0.594 on external benchmarks, recovering the performance of long-standing classical scoring functions such as X-Score and AutoDock Vina from a single architecture-light surface descriptor. Component ablation and TreeSHAP attribution localise the dominant contribution to the heat-kernel signature, particularly at short and mid-range diffusion times that probe local curvature, cavity dimension and global enclosure.

The same descriptor, when injected into a SchNet-style distance-based graph neural network through global concatenation, per-atom projection, DDG-conditional cross-attention or curvature-conditioned edge features, produces only marginal and statistically non-significant lifts on cluster-disjoint testing, with a mean lift of approximately 0.004. Under the present compact backbone and these four integration strategies, the explicit injection of the descriptor therefore did not yield a measurable improvement. This negative result is consistent with the geometric content being already captured by distance-based message passing on atomic coordinates, but it may also reflect the limited capacity and training budget of the backbone or the particular fusion schemes tested, and we accordingly frame it as specific to this architectural setting rather than as a general property of three-dimensional networks.

Taken together these findings define a clear practical and conceptual boundary. Pocket-surface discrete differential geometry is most valuable in lightweight, fingerprint or sequence-based pipelines common in early-stage virtual screening and in resource-limited settings, where it functions as an interpretable, leakage-robust and computationally efficient feature class. For the SchNet-style distance-based graph neural network we tested, the residual benefit of explicit injection is limited. The most promising direction we identify is the development of hybrid mesh-to-atom attention mechanisms, in which ligand atoms attend directly to the rich spectral and curvature information of the pocket surface, and which would allow larger and more diverse geometric deep learning models to exploit the full informational content of the binding cleft as a two-manifold object. All processed data, computed descriptors, trained models and code are openly released to facilitate reproduction and further methodological development.

## Figures and Tables

**Table 1:** Pearson correlation on the cluster-disjoint test fold (t=0.4, n=643). The Δr row reports the lift induced by adding the 59-dimensional discrete differential geometry block to each baseline. The +DDG (residualised) row reports the lift after each DDG channel has been residualised against the surface area channel.

Model	Size	Desc	Morgan	3-mer	Concat
base	0.552	0.560	0.466	0.288	0.468
+DDG	0.622	0.604	0.578	0.534	0.578
Δr	+0.070	+0.044	+0.111	+0.246	+0.111
+DDG (resid.)	0.628	0.608	0.570	0.550	0.584

**Table 2:** Ablation of discrete differential geometry sub-blocks on the cluster-disjoint test fold (t=0.4). Each sub-block is added to the concat baseline in isolation; the combined block is reported in the final row.

DDG Sub-Block (Added to Concat)	Dim	Δr
None (concat alone)	0	-
Curvature ($H,K,\kappa_{1},\kappa_{2},s$)	30	+0.095
Heat-kernel signature ($t_{0}$ to $t_{9}$)	10	+0.117
Laplace–Beltrami eigenvalues	16	+0.093
Mesh statistics	3	+0.094
All sub-blocks combined	59	+0.110

**Table 3:** External benchmark comparison. For the three models developed in this work, we report the scoring power Pearson correlation *r*, the Spearman rank correlation ρ and the Kendall rank correlation τ on the CASF-2016 core set (we evaluate $n_{\mathrm{test}}=253$ of 285) and on the LP-PDBBind DataSAIL S2 leak-proof partition (we evaluate $n_{\mathrm{test}}=626$ of 709). For the literature methods the published scoring power Pearson correlation on the full benchmarks is shown; rank-based coefficients are not uniformly reported across these studies and are left blank. RTMScore is a residue-atom distance-likelihood scoring function optimised for docking and screening power, whose scoring and ranking power on CASF-2016 is reported by its developers as well below the benchmark average, so no scoring power value is tabulated. The full per-configuration metric tables with all three coefficients and 95% bootstrap confidence intervals are provided in Table A3 and Table A4 of Appendix C. Parity plots for the three best-performing CASF-2016 configurations are shown in Figure A12 of the same appendix, and a paired-dot summary of the lift per baseline on CASF-2016 is provided in Figure A13.

	CASF-2016			LP-PDBBind S2		
Method	*r*	*ρ*	*τ*	*r*	*ρ*	*τ*
Ours, concat (base)	0.253	0.263	0.188	0.129	0.136	0.090
Ours, concat plus DDG	0.618	0.624	0.441	0.387	0.382	0.262
Ours, DDG-only	0.594	0.604	0.425	0.456	0.444	0.310
X-Score [5]	0.604	–	–	0.430	–	–
AutoDock Vina [4]	0.604	–	–	0.460	–	–
Pafnucy [32]	0.781	–	–	0.450	–	–
OnionNet-2 [33]	0.821	–	–	–	–	–
IGN [8]	0.837	–	–	0.490	–	–
GIGN [9]	0.840	–	–	0.510	–	–
EHIGN [10]	0.846	–	–	0.520	–	–
RTMScore [34]	docking/screening-oriented			–	–	–

**Table 4:** Four mechanisms for injecting discrete differential geometry information into a SchNet-style atom-level graph neural network, evaluated on the random and cluster-disjoint (t=0.4) splits. Mechanism B.2 concatenates the 59-dimensional descriptor at the readout; B.3.1 projects the per-atom geometric slice into the embedding before message passing; B.3.2 implements DDG-conditional cross-attention in which ligand atoms attend to pocket atoms with attention scores biased by per-atom DDG cosine similarity; B.3.3 augments edge features with Gaussian-expanded $|H_{i}-H_{j}|$, $|\kappa_{1,i}-\kappa_{1,j}|$ and HKS cosine distance in the spirit of CurvAGN.

Split	Configuration	*r*	Δr
Random	atom-only (B.2 baseline)	0.683	-
Random	+ global DDG concat (B.2)	0.687	+0.004
Random	+ per-atom DDG projection (B.3.1)	0.698	+0.018
Random	atom-only (B.3.2 baseline)	0.680	-
Random	+ DDG cross-attention (B.3.2)	0.658	−0.021
Random	atom-only (B.3.3 baseline)	0.682	-
Random	+ curvature-conditioned edges (B.3.3)	0.676	−0.006
Cluster	atom-only (B.2 baseline)	0.669	-
Cluster	+ global DDG concat (B.2)	0.665	−0.004
Cluster	+ per-atom DDG projection (B.3.1)	0.668	+0.011
Cluster	atom-only (B.3.2 baseline)	0.657	-
Cluster	+ DDG cross-attention (B.3.2)	0.674	+0.016
Cluster	atom-only (B.3.3 baseline)	0.668	-
Cluster	+ curvature-conditioned edges (B.3.3)	0.662	−0.006

**Figure A12:**
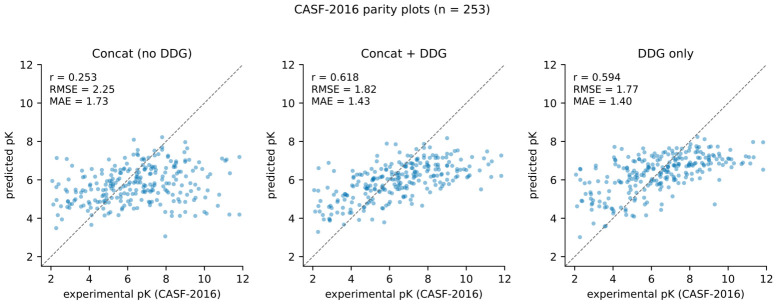
CASF-2016 parity plots for the three best-performing configurations: concat baseline without DDG, concat plus DDG and DDG-only.

**Figure A13:**
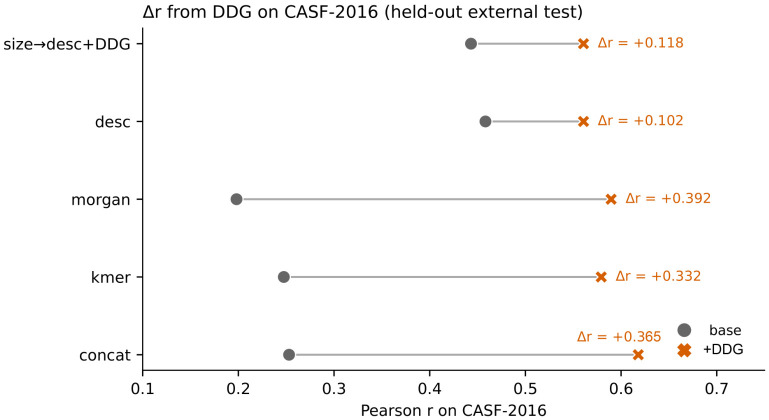
Paired-dot summary of the lift induced by the discrete differential geometry block per baseline on CASF-2016. All lifts are positive and substantial, with the morgan and concat baselines showing the largest values at +0.39 and +0.37 respectively.

## Data Availability

The PDBBind v2020 structures were retrieved from the public Hugging Face mirrors jglaser/pdbbind_complexes and THU-ATOM/PDBbind; the CASF-2016 affinity labels and DataSAIL-curated LP-PDBBind splits were obtained from Zenodo records 8091220 and 17376012.

## Abbreviations

The following abbreviations are used in this manuscript:

DDG	Discrete differential geometry
GBT	Gradient-boosted trees
GNN	Graph neural network
HKS	Heat-kernel signature
LA-SES	Ligand-aware solvent-excluded surface
LP-PDBBind	Leak-proof PDBBind
MAE	Mean absolute error
MD	Molecular dynamics
RMSE	Root-mean-square error
SHAP	Shapley additive explanations

## Appendix A. Dataset Details and Filtration Log

Table A1 summarises the filtration steps that produced the analysis set of 3285 complexes from the raw mirrors, and the corresponding subset coverage for the CASF-2016 core set and the LP-PDBBind DataSAIL S2 leak-proof partition. The empirical cumulative distribution of the cluster size after *k*-mer Jaccard greedy single-linkage clustering at threshold t=0.4 is plotted in Figure A1. The size-bias baseline parity from the five-fold cross-validated linear regressor on heavy-atom count is plotted in Figure A2, and the parity grid covering all five baseline feature sets across the three internal split regimes is reproduced in Figure A3.

**Table A1:** Dataset filtration steps from raw mirrors to the final analysis set used in the main text.

Step	Count
PDBBind v2020 jglaser/pdbbind_complexes (raw)	16,079
∩ BindingDB-derived affinity (joined on sequence and canonical SMILES)	3590
∩ pK ∈[2,11], sequence length ∈[20,2000], unique pdb_id	3558
∩ DDG mesh and features successfully computed	3285
CASF-2016 [6] total	285
∩ retrievable from THU-ATOM/PDBbind mirror in parseable form	253
LP-PDBBind DataSAIL S2 test partition [20]	709
∩ structures retrieved and DDG computed	626

## Appendix B. DDG Feature Distributions and Per-Component Analyses

This appendix collects the dataset-wide distributions of the discrete differential geometry features and the per-component analyses summarised in the main text. The kernel density estimates of the per-vertex curvature features are shown in Figure A4, and the dataset-wide heat-kernel signature spectrum is shown in Figure A5. Hexbin density plots of pK against the four most informative single features are provided in Figure A6. The marginal contribution of the geometric block to each baseline across the three internal split regimes is summarised in Figure A7, and the surface-area partial-out control is presented in Figure A8. Parity plots that compare the base versus augmented configurations on the cluster-disjoint test fold are presented in Figure A9. Bootstrap confidence intervals for each DDG sub-block configuration on both the random and cluster-disjoint splits are presented in Figure A10, and an alternative compact representation of the per-sub-block lift is shown in Figure A11.

## Appendix C. Full Per-Configuration Metric Tables and Parity Plots

This appendix reports the full per-configuration metric tables on the cluster-disjoint test fold and on the two external benchmarks, together with parity plots and the per-baseline lift summary on CASF-2016. The cluster-disjoint full table with 95% bootstrap confidence intervals is provided in Table A2, the CASF-2016 full table in Table A3 and the LP-PDBBind DataSAIL S2 full table in Table A4. Parity plots for the three best CASF-2016 configurations are shown in Figure A12, and a paired-dot summary of the per-baseline lift on CASF-2016 is presented in Figure A13.

**Table A2:** Cluster-disjoint split (Jaccard t=0.4, $n_{\mathrm{test}}=643$). Pearson correlation *r*, Spearman rank correlation ρ and Kendall rank correlation τ, each with a 95% bootstrap confidence interval from 500 resamples. The root-mean-square error and mean absolute error are in pK units.

Feature Set	*r* (95% CI)	ρ (95% CI)	τ (95% CI)	RMSE	MAE
size (LR)	0.551 [0.496, 0.604]	0.552 [0.493, 0.608]	0.391 [0.347, 0.438]	1.47	1.18
desc (GBT)	0.559 [0.506, 0.609]	0.533 [0.477, 0.590]	0.372 [0.329, 0.415]	1.45	1.17
morgan (GBT)	0.466 [0.400, 0.523]	0.452 [0.387, 0.508]	0.311 [0.264, 0.352]	1.54	1.26
3-mer (GBT)	0.290 [0.231, 0.362]	0.303 [0.242, 0.377]	0.204 [0.162, 0.254]	1.67	1.36
concat (GBT)	0.467 [0.407, 0.530]	0.446 [0.378, 0.512]	0.306 [0.258, 0.357]	1.54	1.26
size+DDG (GBT)	0.621 [0.572, 0.671]	0.590 [0.533, 0.644]	0.419 [0.377, 0.460]	1.37	1.09
desc+DDG (GBT)	0.602 [0.554, 0.651]	0.570 [0.515, 0.621]	0.404 [0.362, 0.444]	1.40	1.11
morgan+DDG (GBT)	0.575 [0.522, 0.629]	0.555 [0.496, 0.613]	0.392 [0.347, 0.438]	1.43	1.14
3-mer+DDG (GBT)	0.532 [0.479, 0.581]	0.517 [0.458, 0.573]	0.357 [0.314, 0.400]	1.48	1.19
concat+DDG (GBT)	0.577 [0.525, 0.628]	0.564 [0.509, 0.621]	0.398 [0.356, 0.442]	1.42	1.14
DDG-only (GBT)	0.515 [0.454, 0.577]	0.496 [0.435, 0.556]	0.344 [0.300, 0.388]	1.51	1.20

**Table A3:** CASF-2016 external benchmark ($n_{\mathrm{test}}=253$ of 285; $n_{\mathrm{train}}=3215$). Pearson correlation *r*, Spearman rank correlation ρ and Kendall rank correlation τ, each with a 95% bootstrap confidence interval from 500 resamples; root-mean-square error and mean absolute error in pK units.

Feature Set	*r* (95% CI)	ρ (95% CI)	τ (95% CI)	RMSE	MAE
size (LR)	0.447 [0.345, 0.540]	0.446 [0.337, 0.541]	0.310 [0.230, 0.383]	1.96	1.60
desc (GBT)	0.460 [0.363, 0.552]	0.433 [0.321, 0.532]	0.301 [0.222, 0.377]	1.95	1.57
morgan (GBT)	0.194 [0.067, 0.339]	0.215 [0.088, 0.354]	0.157 [0.072, 0.251]	2.36	1.80
3-mer (GBT)	0.247 [0.139, 0.358]	0.246 [0.125, 0.351]	0.166 [0.085, 0.241]	2.17	1.77
concat (GBT)	0.248 [0.120, 0.375]	0.263 [0.140, 0.387]	0.188 [0.103, 0.273]	2.27	1.74
desc+DDG (GBT)	0.562 [0.470, 0.640]	0.541 [0.437, 0.627]	0.378 [0.302, 0.450]	1.86	1.50
morgan+DDG (GBT)	0.589 [0.519, 0.666]	0.582 [0.504, 0.667]	0.413 [0.351, 0.485]	1.87	1.43
3-mer+DDG (GBT)	0.578 [0.506, 0.659]	0.575 [0.500, 0.657]	0.402 [0.346, 0.468]	1.83	1.46
concat+DDG (GBT)	0.617 [0.548, 0.685]	0.624 [0.551, 0.696]	0.441 [0.386, 0.502]	1.83	1.43
DDG-only (GBT)	0.594 [0.527, 0.666]	0.604 [0.530, 0.679]	0.425 [0.369, 0.489]	1.78	1.41

**Table A4:** LP-PDBBind DataSAIL S2 leak-proof external benchmark ($n_{\mathrm{test}}=626$ of 709; $n_{\mathrm{train}}=3212$). Pearson correlation *r*, Spearman rank correlation ρ and Kendall rank correlation τ, each with a 95% bootstrap confidence interval from 500 resamples; root-mean-square error and mean absolute error in pK units.

Feature Set	*r* (95% CI)	ρ (95% CI)	τ (95% CI)	RMSE	MAE
size (LR)	−0.023 [−0.096, 0.062]	0.075 [−0.003, 0.161]	0.056 [0.003, 0.114]	2.50	1.90
desc (GBT)	0.327 [0.257, 0.395]	0.341 [0.267, 0.410]	0.229 [0.178, 0.278]	1.87	1.47
morgan (GBT)	0.077 [0.004, 0.149]	0.069 [−0.005, 0.141]	0.047 [−0.002, 0.096]	1.99	1.57
3-mer (GBT)	0.162 [0.103, 0.221]	0.161 [0.090, 0.235]	0.105 [0.058, 0.153]	1.88	1.49
concat (GBT)	0.129 [0.063, 0.189]	0.136 [0.062, 0.201]	0.090 [0.040, 0.134]	1.97	1.53
desc+DDG (GBT)	0.428 [0.368, 0.488]	0.438 [0.369, 0.506]	0.300 [0.252, 0.349]	1.69	1.33
morgan+DDG (GBT)	0.387 [0.312, 0.459]	0.364 [0.290, 0.439]	0.257 [0.203, 0.310]	1.74	1.36
3-mer+DDG (GBT)	0.389 [0.322, 0.454]	0.387 [0.317, 0.456]	0.263 [0.212, 0.311]	1.74	1.36
concat+DDG (GBT)	0.389 [0.323, 0.452]	0.382 [0.314, 0.452]	0.262 [0.215, 0.311]	1.74	1.37
DDG-only (GBT)	0.459 [0.395, 0.522]	0.444 [0.371, 0.513]	0.310 [0.257, 0.361]	1.67	1.31

## Appendix D. Case Study: 5u7j (Best) vs. 5yp6 (Worst Predicted)

The two complexes were auto-selected from the cluster-disjoint test fold predictions of the concat plus DDG model as the smallest and the largest absolute residuals, restricted to sequence-length 100 to 500 and pK range 4 to 10 to avoid peptide-like outliers. The kinase-like target 5u7j attains a residual of +0.001pK (predicted 6.41, experimental 6.41), while 5yp6 attains −3.79pK (predicted 8.39, experimental 4.60), a substantial overestimate. The LA-SES surfaces of the two complexes, coloured by per-vertex mean curvature, Gaussian curvature and the heat-kernel signature at $t_{5}$, are shown in the main text in Figure 12. Figure A14 presents the per-ligand-atom geometric profile as a within-ligand *z*-score heatmap, and Figure A15 reports the per-vertex distributions of mean curvature, Gaussian curvature, shape index and HKS at $t_{5}$ for the two pockets.

## Appendix E. Robustness Sensitivity Analyses

This appendix compiles the robustness sensitivity analyses referenced in the main text. The Pearson lift induced by the discrete differential geometry block is plotted in Figure A16 as a function of the cluster Jaccard threshold, showing the monotone increase as the cluster-disjointness becomes stricter. Table A5 summarises the full robustness panel, including the five-seed ensemble means and standard deviations on the cluster-disjoint test fold, the three Jaccard thresholds, the 5000-iteration permutation *p*-value, the time-like alphabetical split lift, and the two external benchmark lifts.

**Table A5:** Robustness summary. Pearson lift across stress tests on both the cluster-disjoint and external benchmarks.

Test	Result
Five-seed ensemble (cluster t=0.4): base *r*	0.512±0.053
Five-seed ensemble (cluster t=0.4): +DDG *r*	0.608±0.068
Five-seed ensemble: Δr	+0.096±0.021
Cluster threshold t=0.3: Δr	+0.129
Cluster threshold t=0.4: Δr	+0.111
Cluster threshold t=0.5: Δr	+0.076
Permutation test (5000 perms): *p*-value	<$2\times 10^{-4}$
Time-like alphabetical split: Δr	+0.068
CASF-2016 external: Δr	+0.365
LP-PDBBind DataSAIL S2: Δr	+0.258

## Appendix F. SchNet-Style GNN Injection: Per-Mechanism Best-Epoch Results

This appendix reports the GNN-side experiments referenced in the main text as B.2 (global DDG concatenation at the readout), B.3.1 (per-atom DDG projection at the embedding stage), B.3.2 (DDG-conditional cross-attention) and B.3.3 (curvature-conditioned edge features in the spirit of CurvAGN). The backbone, training schedule and evaluation protocol are identical across the seven configurations, namely the four DDG-injected variants and three matched atom-only baselines retrained alongside B.2, B.3.2 and B.3.3 to control for stochastic seed effects. All results are single-seed, best-epoch test Pearson correlations on Google Colaboratory NVIDIA T4 GPU. Multi-seed confidence intervals on the graph neural network results were not collected due to compute budget; the reported lifts are best-epoch single-seed differences whose reliability is bounded by the four-mechanism consistency reported in Table A6. The graph neural network parity plots and training curves were generated on Google Colaboratory and are released in the project’s data archive on Zenodo, together with the feature-based pipeline.

**Table A6:** SchNet-style atom-level graph neural network with four DDG injection mechanisms on the random and cluster-disjoint (t=0.4) splits. All entries are single-seed and the Pearson lift Δr is computed against the matched atom-only baseline retrained alongside each variant. The Pearson correlation of approximately 0.68 on the random split is below published large-scale 3D atom-level GNNs at approximately 0.84 on CASF-2016; the small backbone is retained for controlled feature-injection ablation.

Split	Configuration	*r*	Δr	Best Epoch
Random	atom-only (B.2 baseline)	0.683	-	15
Random	+ global DDG concat (B.2)	0.687	+0.004	27
Random	+ per-atom DDG projection (B.3.1)	0.698	+0.018	24
Random	atom-only (B.3.2 baseline)	0.680	-	22
Random	+ DDG cross-attention (B.3.2)	0.658	−0.021	18
Random	atom-only (B.3.3 baseline)	0.682	-	21
Random	+ curvature-conditioned edges (B.3.3)	0.676	−0.006	23
Cluster	atom-only (B.2 baseline)	0.669	-	19
Cluster	+ global DDG concat (B.2)	0.665	−0.004	24
Cluster	+ per-atom DDG projection (B.3.1)	0.668	+0.011	21
Cluster	atom-only (B.3.2 baseline)	0.657	-	26
Cluster	+ DDG cross-attention (B.3.2)	0.674	+0.016	25
Cluster	atom-only (B.3.3 baseline)	0.668	-	22
Cluster	+ curvature-conditioned edges (B.3.3)	0.662	−0.006	25

## Appendix G. Software and Hardware Versions

The complete list of software versions used in the analysis pipeline is provided in Table A7. The feature-based experiments, including the gradient-boosted tree training, ablation, permutation test, five-seed ensemble and the external benchmark evaluations on CASF-2016 and LP-PDBBind, ran on a single Apple Silicon laptop with an M-series central processing unit. The discrete differential geometry batch processing and the graph neural network training ran on Google Colaboratory with an NVIDIA T4 graphics processing unit with 16 gigabytes of memory. End-to-end runtime was approximately 36 min for the 3285-complex DDG batch on Colab T4, approximately two minutes per 30-epoch graph neural network seed, and at most five minutes for any individual feature-based fit-evaluate-bootstrap cycle.

**Table A7:** Software versions used in the analysis pipeline.

Component	Version
Python	3.10
NumPy	1.26
Pandas	2.2
PyArrow	24.0
SciPy	1.13
scikit-learn	1.4.2
RDKit	2024.03
trimesh	4.12.2
scikit-image	0.25.2
libigl-python (igl)	2.5+
biopython	1.87
matplotlib	3.10
seaborn	0.13.2
PyTorch	2.3 (Colab T4 only)
torch-geometric	2.5.3 (Colab T4 only)

**Table A8:** Key mesh-generation parameters, gradient-boosted tree hyperparameters and exact random seeds used throughout the analysis. Unlisted tree settings use the scikit-learn 1.4 defaults.

Setting	Value
Ligand-aware solvent-excluded surface and DDG descriptor	
Pocket cutoff	protein heavy atoms within 10 Å of any ligand heavy atom
Probe radius	1.4 Å
Signed-distance grid resolution	0.8 Å (1.12 Å for very large bounding boxes)
Surfacing	marching cubes, largest connected component retained
Smoothing	8 Taubin iterations (λ=0.5, μ=−0.53)
Decimation	quadric-error-metric edge collapse to ≤12,000 faces
Resulting mesh size	≈6000 vertices, ≈12,000 faces
Laplace–Beltrami operator	cotangent Laplacian; leading 16 eigenvalues
Heat-kernel signature	10 logarithmically spaced diffusion times
Curvature moments	mean, std, 10th and 90th percentile, skew, kurtosis
Descriptor dimension	59 (30 curvature + 16 eigenvalues + 10 HKS + 3 mesh)
Gradient-boosted tree (HistGradientBoostingRegressor)	
Loss	Huber
Learning rate	0.05
Maximum tree depth	6
Maximum iterations	300 (≤100 features), 200 (>100 features)
Early stopping	disabled
Size-only baseline	ordinary linear regression
Random seeds	
Random split	42
Target-disjoint split	43
Cluster-disjoint split	44
Five-seed ensemble	cluster-split seeds 44–48, booster seeds 0–4
Graph neural network	42
Bootstrap resampling	500 resamples; permutation test 5000 iterations
